# Complete Genome Sequencing of Acinetobacter baumannii AC1633 and Acinetobacter nosocomialis AC1530 Unveils a Large Multidrug-Resistant Plasmid Encoding the NDM-1 and OXA-58 Carbapenemases

**DOI:** 10.1128/mSphere.01076-20

**Published:** 2021-01-27

**Authors:** Ahmed Ghazi Alattraqchi, Farahiyah Mohd Rani, Nor Iza A. Rahman, Salwani Ismail, David W. Cleary, Stuart C. Clarke, Chew Chieng Yeo

**Affiliations:** a Faculty of Medicine, Universiti Sultan Zainal Abidin, Kuala Terengganu, Terengganu, Malaysia; b Faculty of Medicine and Institute for Life Sciences, University of Southampton, Southampton, United Kingdom; c NIHR Southampton Biomedical Research Centre, University Hospital Southampton NHS Trust, Southampton, United Kingdom; d Global Health Research Institute, University of Southampton, Southampton, United Kingdom; e School of Postgraduate Studies, International Medical University, Kuala Lumpur, Malaysia; f Centre for Translational Research, IMU Institute for Research, Development and Innovation (IRDI), Kuala Lumpur, Malaysia; Antimicrobial Development Specialists, LLC

**Keywords:** NDM-1, OXA-58, carbapenem resistance, Tn125, p*dif* modules, *Acinetobacter baumannii*, *Acinetobacter nosocomialis*, plasmids

## Abstract

Carbapenem-resistant Acinetobacter spp. are considered priority drug-resistant human-pathogenic bacteria. The genomes of two carbapenem-resistant Acinetobacter spp. clinical isolates obtained from the same tertiary hospital in Terengganu, Malaysia, namely, A. baumannii AC1633 and A. nosocomialis AC1530, were sequenced. Both isolates were found to harbor the carbapenemase genes *bla*_NDM-1_ and *bla*_OXA-58_ in a large (ca. 170 kb) plasmid designated pAC1633-1 and pAC1530, respectively, that also encodes genes that confer resistance to aminoglycosides, sulfonamides, and macrolides. The two plasmids were almost identical except for the insertion of IS*Aba11* and an IS*4* family element in pAC1633-1, and IS*Aba11* along with *relBE* toxin-antitoxin genes flanked by inversely orientated p*dif* (XerC/XerD) recombination sites in pAC1530. The *bla*_NDM-1_ gene was encoded in a Tn*125* composite transposon structure flanked by IS*Aba125*, whereas *bla*_OXA-58_ was flanked by IS*Aba11* and IS*Aba3* downstream and a partial IS*Aba3* element upstream within a p*dif* module. The presence of conjugative genes in plasmids pAC1633-1/pAC1530 and their discovery in two distinct species of Acinetobacter from the same hospital are suggestive of conjugative transfer, but mating experiments failed to demonstrate transmissibility under standard laboratory conditions. Comparative sequence analysis strongly inferred that pAC1633-1/pAC1530 was derived from two separate plasmids in an IS*1006*-mediated recombination or transposition event. A. baumannii AC1633 also harbored three other plasmids designated pAC1633-2, pAC1633-3, and pAC1633-4. Both pAC1633-3 and pAC1633-4 are cryptic plasmids, whereas pAC1633-2 is a 12,651-bp plasmid of the GR8/GR23 Rep3-superfamily group that encodes the *tetA*(*39*) tetracycline resistance determinant in a p*dif* module.

**IMPORTANCE** Bacteria of the genus Acinetobacter are important hospital-acquired pathogens, with carbapenem-resistant A. baumannii listed by the World Health Organization as the one of the top priority pathogens. Whole-genome sequencing of carbapenem-resistant A. baumannii AC1633 and A. nosocomialis AC1530, which were isolated from the main tertiary hospital in Terengganu, Malaysia, led to the discovery of a large, ca. 170-kb plasmid that harbored genes encoding the New Delhi metallo-β-lactamase-1 (NDM-1) and OXA-58 carbapenemases alongside genes that conferred resistance to aminoglycosides, macrolides, and sulfonamides. The plasmid was a patchwork of multiple mobile genetic elements and comparative sequence analysis indicated that it may have been derived from two separate plasmids through an IS*1006*-mediated recombination or transposition event. The presence of such a potentially transmissible plasmid encoding resistance to multiple antimicrobials warrants vigilance, as its spread to susceptible strains would lead to increasing incidences of antimicrobial resistance.

## Abstract (precis)

Bacteria of the genus Acinetobacter are important hospital-acquired pathogens, with carbapenem-resistant A. baumannii listed by the World Health Organization as the one of the top priority pathogens. Whole-genome sequencing of carbapenem-resistant A. baumannii AC1633 and A. nosocomialis AC1530, which were isolated from the main tertiary hospital in Terengganu, Malaysia, led to the discovery of a large, ca. 170-kb plasmid that harbored genes encoding the New Delhi metallo-β-lactamase-1 (NDM-1) and OXA-58 carbapenemases alongside genes that conferred resistance to aminoglycosides, macrolides, and sulfonamides.

## INTRODUCTION

Infections caused by the Gram-negative pathogen Acinetobacter baumannii have become increasingly problematic, particularly among immunocompromised patients and patients in intensive care units, due to its ability to acquire and develop resistance to multiple antimicrobials and thereby severely limit treatment options (1, 2). The genomes of Acinetobacter strains are flexible and adaptable, prone to accumulating antibiotic resistance determinants through horizontal gene transfer involving mobile genetic elements (3, 4). Resistance to carbapenems, which are among the antimicrobials of last resort for the treatment of multidrug-resistant (MDR) Acinetobacter infections, is increasing, with resistance rates exceeding 90% in certain regions of the world (5). Of pressing concern, pandrug-resistant (PDR) isolates of A. baumannii, which are resistant to all classes of antimicrobials, have been reported from clinical as well as environmental samples (6–8). In the CDC’s 2019 Antibiotic Resistance Threats Report, carbapenem-resistant A. baumannii has been listed as an “urgent” threat (9). Likewise, the World Health Organization (WHO) has categorized carbapenem-resistant A. baumannii as a critical priority pathogen toward which new antimicrobials are urgently needed (10).

A. nosocomialis is closely related to A. baumannii and, along with A. pittii, A. seifertii, A. dijkshoorniae, and A. calcoaceticus, they are often grouped together as the A. baumannii-A. calcoaceticus (Abc) complex due to difficulties in identifying these bacteria by traditional biochemical methods (11, 12). In our recent study of Acinetobacter isolates obtained from the main tertiary hospital in the state of Terengganu, Malaysia in 2015, the majority (83.7%) were A. baumannii followed by A. nosocomialis (10.4%), with multidrug resistance much more prevalent in A. baumannii (13). Nevertheless, A. nosocomialis and other members of the Abc complex are clinically relevant, with carbapenem-resistant and MDR isolates being reported (14).

Multiple mechanisms of drug resistance are usually at play in Acinetobacter isolates and these include enzymatic inactivation of the antibiotic, modifications in the target sites, reduced accumulation of antibiotics through expression of efflux systems or mutations in outer membrane channels, and the formation of biofilms (15, 16). Carbapenem resistance in Acinetobacter is frequently attributed to the acquisition and production of OXA β-lactamases, which are categorized as Ambler class D enzymes with a serine residue within the active site that catalyzes the hydrolysis of the β-lactam substrate (17). Common acquired OXA subtypes found in Acinetobacter include OXA-23, OXA-24/40, OXA-58, OXA-143, and OXA-235, with the genes encoding them usually associated with or located in mobile genetic elements (16, 17). In some instances, an upstream and adjacent insertion sequence (IS) element provides a strong outward-directing promoter for their expression (17). A. baumannii also harbors an intrinsic *bla*_OXA-51_/*bla*_OXA-51-like_ gene in its chromosome and, although the OXA-51/OXA-51-like enzyme has been shown to hydrolyze imipenem and meropenem, its affinity for these carbapenems is quite low and would not normally confer carbapenem resistance (17). However, the insertion of IS elements with outward-directing promoters, such as IS*Aba1*, upstream of the *bla*_OXA-51_/*bla*_OXA-51-like_ gene has been shown to increase expression of the enzyme, leading to carbapenem resistance (18). Nevertheless, recent reports have indicated that in the absence of an acquired carbapenemase gene, the presence of IS*Aba1* or similar elements upstream and adjacent to the intrinsic *bla*_OXA-51_/*bla*_OXA-51-like_ gene does not always guarantee carbapenem resistance in these isolates (13, 19).

The metallo-β-lactamases (MBLs) or Ambler class B enzymes, such as the New Delhi metallo-β-lactamase (NDM) group, represents another class of acquired carbapenemases that have been found in Acinetobacter spp., being first reported in A. baumannii from India (20) and China (21). MBLs, including NDMs, are dependent on zinc ions at the active site of the enzyme (16). The *bla*_NDM-1_ gene has since been found in many other Acinetobacter spp., is usually carried in the composite transposon Tn*125* or its derivatives, and is either plasmid- or chromosome-encoded (22). NDM-1 confers resistance to all β-lactams except monobactams, such as aztreonam, and is not inhibited by β-lactamase inhibitors such as clavulanic acid, sulbactam, tazobactam, and avibactam (16, 23, 24). Most isolates that harbor the *bla*_NDM-1_ gene are likely MDR or extensive-drug resistant (XDR) due to the association of *bla*_NDM-1_ with other resistance genes (16, 22).

Here, we report the whole-genome sequences of two NDM-1-producing, carbapenem-resistant Acinetobacter clinical isolates, A. baumannii AC1633 and A. nosocomialis AC1530, obtained from the main tertiary hospital in the state of Terengganu in Peninsular Malaysia. We show that in these two isolates, the *bla*_NDM-1_ gene is colocated with *bla*_OXA-58_ on a large ca. 170-kb plasmid along with various other antimicrobial-resistance genes, and that the carriage of this plasmid in these two isolates likely led to their MDR status. We also show sequence evidence that this plasmid was likely derived from two plasmids that separately encoded *bla*_NDM-1_ and *bla*_OXA-58_ in a Malaysian A. pittii isolate and that they combined via an IS*1006*-mediated recombination event.

## RESULTS AND DISCUSSION

### Background of the A. baumannii AC1633 and A. nosocomialis AC1530 clinical isolates.

A. baumannii AC1633 and A. nosocomialis AC1530 are part of our collection of Acinetobacter spp. clinical isolates that were obtained since 2011 from Hospital Sultanah Nur Zahirah (HSNZ), the main public tertiary hospital in the state of Terengganu, Malaysia (13, 25). Whole-genome sequencing was performed on a random selection of 50 isolates obtained from 2011 to 2016 (manuscript in preparation) and preliminary analyses of the genome sequences indicated two isolates that harbored the *bla*_NDM-1_ gene, i.e., AC1633 and AC1530.

A. baumannii AC1633 was isolated from the blood of a 60-year-old female patient in the neurology intensive care unit in April 2016. The patient had hospital-acquired pneumonia with respiratory failure and eventually succumbed to septicemia 41 days after hospital admission. A. nosocomialis AC1530 was isolated from the blood of a 14-year-old male patient in the surgical ward in April 2015. The patient was admitted for polytrauma due to a motor vehicle accident and developed hospital-acquired pneumonia complicated with right parapneumonic effusion, but recovered and was discharged after 60 days. A. baumannii AC1633 was resistant to the carbapenems (with imipenem, meropenem, and doripenem MIC values of >32 μg/ml each), cephalosporins (cefotaxime, ceftriaxone, ceftazidime, and cefepime), β-lactam/β-lactamase inhibitor combinations (piperacillin-tazobactam and ampicillin-sulbactam), trimethoprim-sulfamethoxazole, ciprofloxacin, and tetracycline. AC1633 also showed resistance to gentamicin but was susceptible to the other aminoglycosides tested (namely, amikacin and tobramycin), as well as to levofloxacin, doxycycline, and the polymyxins (polymyxin B and colistin). A. nosocomialis AC1530 was resistant to the carbapenems (with MIC values for imipenem, meropenem, and doripenem of >32 μg/ml each), cephalosporins (cefotaxime, ceftriaxone, ceftazidime, and cefepime), trimethoprim-sulfamethoxazole, and gentamicin, but was susceptible to all other antibiotics tested. Thus, both AC1633 and AC1530 are categorized as MDR following the criteria proposed by the joint commission of the U.S. Centers for Disease Control and Prevention (CDC) and the European Centre for Disease Prevention and Control (ECDC) (26).

### Whole-genome sequencing and comparative analyses of AC1633 and AC1530.

Analysis of the Illumina-sequenced genomes of A. baumannii A1633 and A. nosocomialis AC1530 indicated the presence of the *bla*_NDM-1_ and *bla*_OXA-58_ carbapenemase genes. Production of the NDM-1 metallo-β-lactamase (MBL) in both isolates was validated by testing with the Etest MBL kit (bioMérieux). Further analyses of the assembled genome data of AC1530 and AC1633 revealed the possibility that the *bla*_NDM-1_ and *bla*_OXA-58_ genes could be harbored in either one or two large plasmids in both isolates; however, this was difficult to ascertain as there were more than 20 assembled contigs from each isolate’s genome data that could potentially belong to these plasmids. Thus, the genomic DNA of these two isolates was subjected to PacBio sequencing and hybrid assembly was then performed on the PacBio and Illumina reads. The resulting assembled genome features of these two isolates are listed in Table 1.

**TABLE 1:** Genome features of A. baumannii AC1633 and A. nosocomialis AC1530

Feature	A. baumannii AC1633					A. nosocomialis AC1530	
	Chromosome	pAC1633-1	pAC1633-2	pAC1633-3	pAC1633-4	Chromosome	pAC1530
Size (bp)	4,364,474	174,292	12,651	9,950	5,210	3,980,182	173,972
% GC content	39	38	36	36	37	38	38
Total no. of genes	2,112	29	4		1	1,918	29
No. of rRNA operons	18					18	
No. of tRNAs	72					74	
Total no. of coding sequences (CDS)	4,096	176	18	13	8	3,750	180

**FIG 1:**
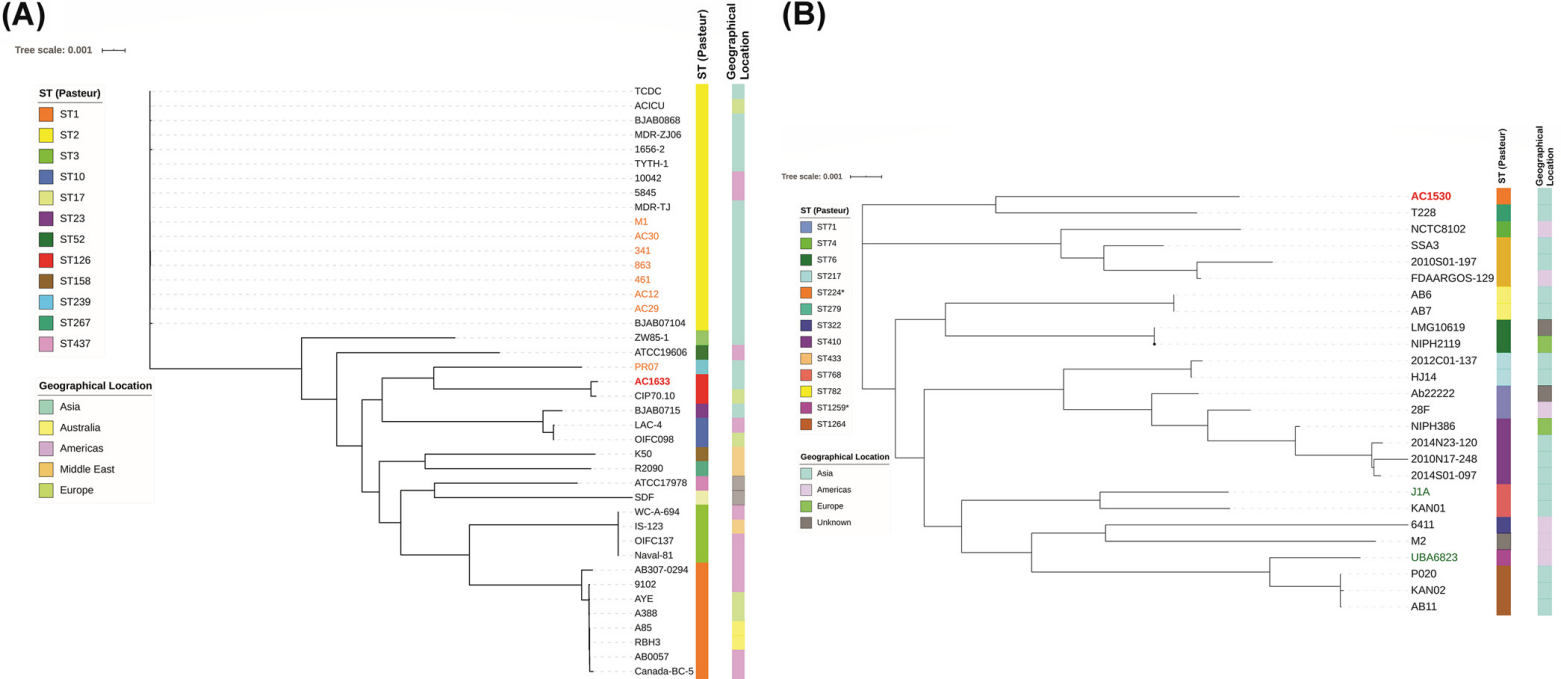
Core genome phylogenetic trees of A. baumannii AC1633 (A) and A. nosocomialis AC1530 (B) in comparison with other related isolates. The A. baumannii core genome comprises 1,167 genes from a total of 20,102 genes, whereas the A. nosocomialis core genome consisted of 1,147 genes from a total of 18,024 genes. The sequence types (STs) of the isolates as determined using the Pasteur scheme are presented by the colored bar on the immediate right of the respective trees. For A. baumannii in (A), ST1 corresponds to the global clone 1 (GC1) lineage, ST2 to GC2 and ST3 to GC3. The geographical location of the respective isolates is also presented as a colored bar on the furthest right of each tree. In (A), A. baumannii isolates from Malaysia are indicated in orange fonts (except AC1633, which is indicated in bold red font) and in (B), A. nosocomialis environmental isolates are indicated in blue-green fonts. All other A. nosocomialis isolates were obtained from clinical samples. Details of the A. baumannii and A. nosocomialis isolates that were used in the phylogenetic analyses are in Table S1 in the supplemental material.

The genome of A. baumannii AC1633 is nearly 4.4 Mb in size and is comprised of a single chromosome of 4.36 Mb (accession no. CP059300) and four plasmids designated pAC1633-1 (174 kb; CP059301), pAC1633-2 (12.6 kb; CP059303), pAC1633-3 (9.9 kb; CP059304), and pAC1633-4 (5.2 kb; CP059302). A. baumannii AC1633 is typed as ST2089 under the Oxford multilocus sequence type (MLST) scheme and ST126 under the Pasteur MLST scheme. AC1633 does not belong to any of the major A. baumannii global clonal lineages, and phylogenetic analysis of the whole-genome sequence in comparison with the sequences of selected A. baumannii isolates (Fig. 1A, Table S1 in the supplemental material) showed it is most closely related to A. baumannii CIP70.10 (ATCC 15151), which was isolated in France in 1970 and is an important reference strain due to its susceptibility to most antimicrobials (27). CIP70.10 also belonged to the same STs as AC1633. Phylogenetic analyses also indicated that AC1633 is not closely related to any of the few Malaysian A. baumannii genomes that are currently available in the database, most of which belonged to the Global Clonal 2 (GC2) lineage (28, 29). A. baumannii isolates AC12, AC29, and AC30, which were isolated from the same hospital as AC1530 and AC1633 but in the year 2011, were of the sequence type ST195 (Oxford)/ST2 (Pasteur) (30, 31). A recent report of 13 A. baumannii genomes from Malaysia indicated three isolates that harbored *bla*_NDM-1_ (29); however, we were unable to compare these isolates with ours as the sequence files that were associated with the GenBank accession numbers provided in the manuscript have yet to be publicly released at the time of writing (December 24, 2020). Using the KAPTIVE database that enables the typing of A. baumannii strains by variation in their composition and structure of capsular polysaccharide (CPS) biosynthetic genes (32), AC1633 was typed as OCL6 for the outer core biosynthesis locus and KL14 for the K locus, which contain genes responsible for the biosynthesis and export of CPS, and both loci were typed with 100% match confidence level.

10.1128/mSphere.01076-20.1 TABLE S1 (A) Characteristics of the Acinetobacter baumannii strains that were used in comparative genomics and phylogenetic tree construction in Fig. 1A and Table S1. (B) Characteristics of the Acinetobacter nosocomialis strains that were used in comparative genomics and phylogenetic tree construction in Fig. 1B. Download Table S1, DOCX file, 0.1 MB. Copyright © 2021 Alattraqchi et al. 2021 Alattraqchi et al. This content is distributed under the terms of the Creative Commons Attribution 4.0 International license.

A. nosocomialis AC1530 has a single chromosome of 3.98 Mb (CP045560.1) and a plasmid of 173.9 kb designated pAC1530 (CP045561.1). AC1530 was assigned by the curators of the PubMLST database (33) to the Pasteur ST1539 (with alleles *cpn60*-47, *fusA*-26, *gltA*-50, *pyrG*-14, *recA*-26, *rplB*-16, and *rpoB*-49) and Oxford ST2195 (with alleles *cpn60*-73, *gdhB*-86, *gltA*-76, *gpi*-4, *gyrB*-65, *recA*-21, and *rpoD*-90). Phylogenetic analyses showed that the closest relative of AC1530 is A. nosocomialis T228 (accession no. JRUA01000001.1), a clinical isolate obtained from Bangkok, Thailand in 2010 (Fig. 1B, Table S1). However, A. nosocomialis T228 was typed as Pasteur ST279 and Oxford ST1897, whereas AC1530 shared only a single allele in the Oxford scheme (*gyrB*-65) and two alleles in the Pasteur scheme (*fusA*-26, *rplB*-16) with T228.

### Antimicrobial resistance genes in the genomes of AC1633 and AC1530.

Interestingly, the bulk of the acquired antimicrobial resistance genes for A. baumannii AC1633 and A. nosocomialis AC1530 came from the large ca. 170-kb plasmid, pAC1633-1 and pAC1530, respectively (Table 2). A. baumannii AC1633 harbored two β-lactam resistance genes in its chromosome, i.e., the intrinsic *bla*_OXA-51-like_ gene categorized as *bla*_OXA-116_ and the Acinetobacter-derived AmpC cephalosporinase (ADC) gene *bla*_ADC-25_ (accession no. EF016355.1) (34). In some cases, carbapenem resistance and increased cephalosporin resistance have been linked with upregulation of the respective *bla*_OXA-51_/*bla*_OXA-51-like_ or *bla*_ADC_ genes through insertion of IS*Aba1* or related IS elements that harbor outward-directing promoters (18, 35, 36); however, no such IS elements could be found upstream of the *bla*_OXA-116_ and *bla*_ADC-25_ genes in A. baumannii AC1633. Tetracycline resistance in AC1633 is likely mediated by the *tetA*(*39*) gene that was carried on the smaller 12.6-kb plasmid, pAC1633-2 (Table 2), and that encodes the tetracycline-specific TetA(39) efflux pump of the major facilitator superfamily (MFS). Notably, pAC1633-1 also harbors the *adeABC* operon that encodes the multidrug resistance-nodulation-cell division (RND) family efflux system along with its two-component regulatory system, *adeRS*, which is located upstream and transcribed divergently from *adeABC*. This efflux system is usually chromosome encoded in Acinetobacter and the multidrug resistance phenotype has been shown to correlate with overexpression of *adeABC* (37, 38). Interestingly, the A. baumannii AC1633 chromosome harbors another copy of *adeRS* and *adeAB* (nucleotides [nt] 1011642 to 1016860), but *adeC* was absent. There were no obvious signs of mobile elements or genomic islands in the vicinity of the chromosomal *adeRS-adeAB* genes that could explain the deletion of *adeC*. The chromosome of AC1633 also harbors genes encoding the other Acinetobacter RND family efflux pumps *adeFGH* and *adeIJK*, along with their respective regulatory genes *adeL* and *adeN* (37, 39), three genes encoding MFS efflux pumps, i.e., *abaF*, *abaQ*, and *amvA*, and, finally, *abeS*, which encodes a small-multidrug resistance (SMR) family efflux pump (Table 3).

**TABLE 2:** Antimicrobial resistance phenotype and carriage of antimicrobial resistance genes and chromosomal gene mutations in A. baumannii AC1633 and A. nosocomialis AC1530

Strain	Antimicrobial resistance phenotype by class/carriage of resistance genes or chromosomal mutations^*b*^						
	Carbapenems	Cephalosporins	Aminoglycosides	Tetracyclines	Fluoroquinolones	Sulfonamides	Macrolides
A. baumannii AC1633	IMI^R^ MEM^R^ DOR^R^	CTX^R^ FOX^R^ TAZ^R^ FEP^R^	GEN^R^ AMI^S^ TOB^S^	TET^R^ DOX^S^	CIP^R^ LEV^S^	SXT^R^	ND
Chromosome	*bla*_OXA116_ (*bla*_OXA-51-like_)	*bla*_ADC-25_			*gyrA* S81L^*a*^, *parC* V104I D105E^*a*^		
pAC1633-1	*bla*_NDM-1,_ *bla*_OXA-58_		*aac*(*3*)-*IId*, *aph*(*3*”)-*Ib*, *aph*(*6*)-*Id*			*sul2*	*msrE*, *mphE*
pAC1633-2				*tetA*(*39*)			
pAC1633-3							
pAC1633-4							
A. nosocomialis AC1530	IMI^R^ MEM^R^ DOR^R^	CTX^R^ FOX^R^ TAZ^R^ FEP^R^	GEN^R^ AMI^S^ TOB^S^	TET^S^ DOX^S^	CIP^S^ LEV^S^	SXT^R^	ND
Chromosome		*bla*_ADC-68_			*parC* V104I D105E^*a*^		
pAC1530	*bla*_NDM-1,_ *bla*_OXA-58_		*aac*(*3*)-*IId*, *aph*(*3*”)-*Ib*, *aph*(*6*)-*Id*			*sul2*	*msrE*, *mphE*

a Mutations in the chromosomally encoded *gyrA* and *parC* genes.

b IMI, imipenem; MEM, meropenem; DOR, doripenem; CTX, ceftriaxone; FOX, cefotaxime; TAZ, ceftazidime; FEP, cefepime; GEN, gentamicin; AMI, amikacin; TOB, tobramycin; TET, tetracycline; DOX, doxycycline; CIP, ciprofloxacin; LEV, levofloxacin; SXT, trimethoprim-sulfamethoxazole; ND, not determined; R, resistance; I, intermediate resistance; S, susceptible.

**TABLE 3:**
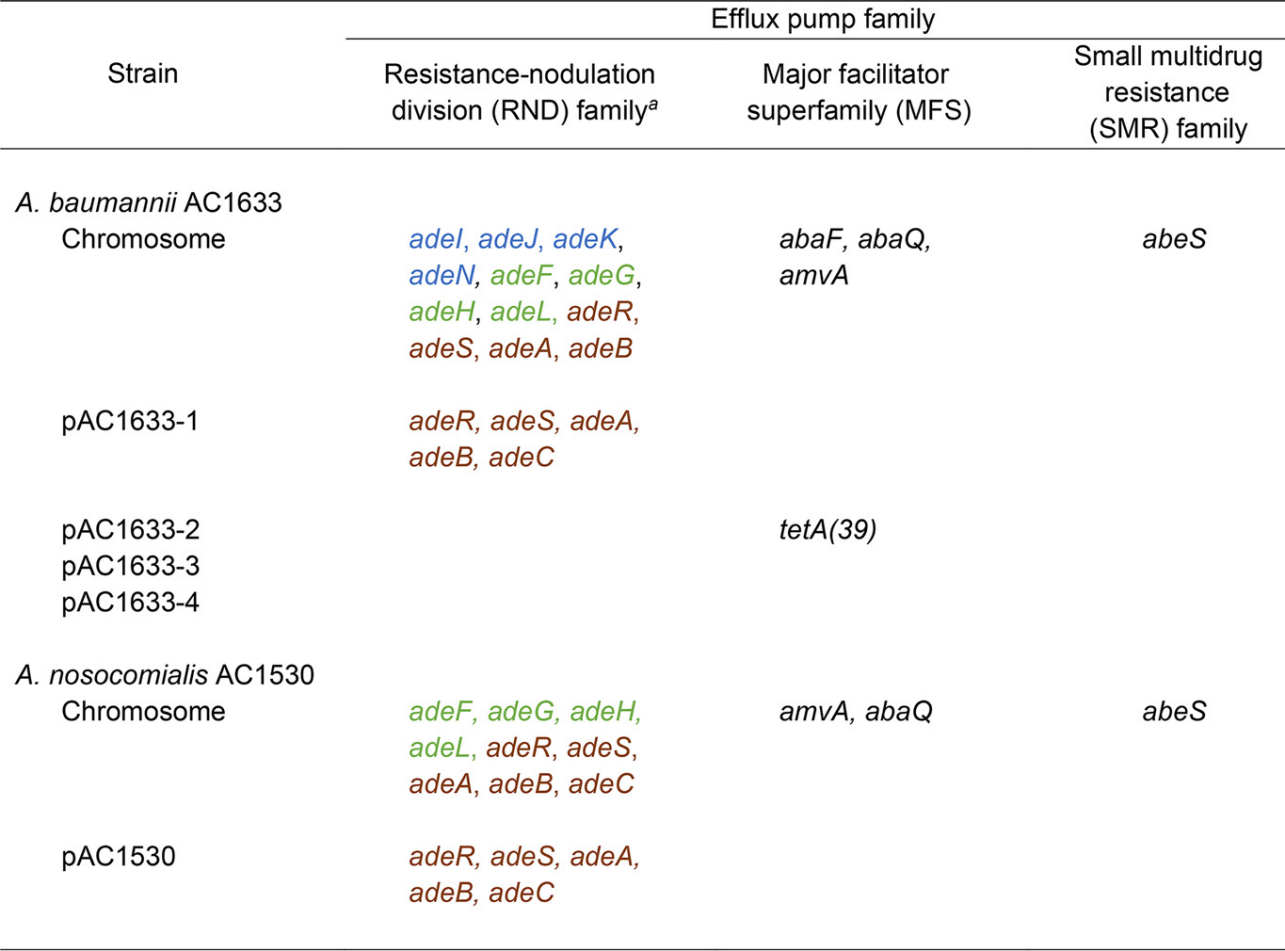
Carriage of efflux-mediated antimicrobial resistance genes in A. baumannii AC1633 and A. nosocomialis AC1530

a Genes in the same colored font indicate that they are part of an operon.

As for A. nosocomialis AC1530, very few antimicrobial resistance genes are found in its chromosome, and the only resistance gene encoding an antibiotic-inactivating enzyme is a *bla*_ADC_-encoding cephalosporinase that shared 94% amino acid sequence identity with ADC-68 (accession no. AGL39360.1) (40). However, as in the A. baumannii AC1633 genome, no IS elements with outward-directing promoters could be detected upstream of this gene. In contrast to AC1633, the chromosome of A. nosocomialis AC1530 harbored a full copy of the *adeRS-adeABC* efflux system besides the copy that resides on pAC1530. Other efflux pumps found encoded in the AC1530 chromosome are *adeFGH* and its regulatory gene *adeL*, the MFS superfamily pumps *amvA* and *abaQ*, and the SMR family *abeS* (Table 3). The same suite of resistance genes in pAC1633-1 was found in pAC1530 (Table 2) and, therefore, likely contributes to its resistance phenotype.

### Characteristics of pAC1633-1 and pAC1530 and their carriage of antimicrobial resistance genes.

**FIG 2:**
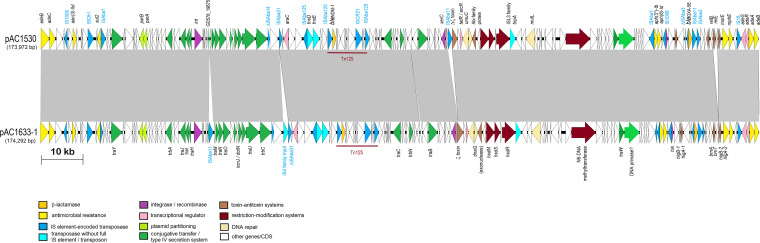
Comparative linear map of plasmids pAC1633-1 and pAC1530. Arrows indicate the extents and directions of genes and ORFs. The *bla*_NDM-1_ and *bla*_OXA-58_ genes are colored in gold, other antimicrobial resistance genes are in yellow. Putative transcriptional activators are in pink, including the genes encoding the two-component regulatory proteins *adeR* and *adeS* that control transcription of the *adeABC* efflux pump. Transposases encoded by full-copy IS elements are shown in dark blue, whereas transposases without their corresponding IS elements or transposons in full are depicted in light blue. Genes with homology to conjugative transfer or type IV secretion system genes are in dark green. GD578_19675 refers to the ORF in the conjugative region of pAC1530 that was the site of insertion for IS*Aba11* in pAC1633-1. Color codes for the other genes are as indicated. Tn*125* that harbors the *bla*_NDM-1_ gene is labeled. The extent of regions with >99% nucleotide sequence identities are indicated in the gray-shaded area.

Plasmids pAC1653-1 from A. baumannii AC1633 and pAC1530 from A. nosocomialis AC1530 were nearly identical except at five regions as follows (Fig. 2): (i) insertion of IS*Aba11* into an open reading frame (ORF) encoding an 85-aa (amino acid) hypothetical protein (locus tag: GD578_19675; accession no. QGA46103.1) which contains a ribosomal protein L7/L12 C-terminal domain (pfam00542) in pAC1530 at nt 40824 (nt 40825 to 41927 of pAC1633-1); this ORF is upstream of the *traMNO* genes and lies within a cluster of genes that are proposed to be part of the conjugative transfer region for the plasmid; (ii) an IS*4* family transposase at nt 58863 to 60133 in pAC1633-1 with no matches to existing IS elements in the ISFinder database (41) and no inverted repeats flanking the putative transposase gene; this is likely a remnant of an IS element that had inserted within IS*Aba31*, leading to only a partial IS*Aba31* downstream of this IS*4*-family remnant element (a full-length IS*Aba31* is found in the corresponding site in pAC1530 with a characteristic 2-bp “TA” direct repeat flanking the IS element); (iii) a 255-bp insertion within a hypothetical ORF at nt 89589 of pAC1530 in pAC1633-1 (nt 91978 to 92233); no characteristic signature sequences of mobile elements could be detected within this short fragment; (iv) insertion of IS*Aba11* into an ORF encoding a putative toxin of the Zeta toxin family in pAC1530; this insertion, at nt 101270 of pAC1633-1, led to a 5-bp direct repeat (TATAG) in pAC1530 (nt 98631 to 99731); and (v) addition of an *relBE* toxin-antitoxin (TA) system along with a downstream ORF encoding a protein of the SMI1/KNR4 family in pAC1530 at nt 164446 of pAC1633-1; this 1,192-bp fragment is flanked by p*dif* (XerD/XerC) recombination sites, more of which will be discussed in a later section. pAC1633-1 and pAC1530 belong to a group of diverse Acinetobacter plasmids that do not have an identifiable replication initiator or replicase (Rep) protein (42, 43).

Both pAC1633-1 and pAC1530 represent a repository of several antimicrobial resistance genes, including the *bla*_NDM-1_ and *bla*_OXA-58_ carbapenemase genes (Table 2). Three aminoglycoside resistance genes were found in both plasmids and all three encoded aminoglycoside-modifying enzymes. The *aac*(*3*)-*IId* group is a subclass of the AAC(3) enzymes that catalyze acetylation of the NH_2_ group at the 3-position of the aminoglycoside 2-deoxystreptamine nucleus and usually confers resistance to gentamicin, sisomicin, and fortimicin (44). Both A. baumannii AC1633 and A. nosocomialis AC1530 were gentamicin resistant, but their susceptibilities against sisomicin and fortimicin were not tested. The other two aminoglycoside resistance genes found in pAC1633-1 and pAC1530 encode the aminoglycoside *O*-phophotransferases (APHs), *aph*(*6*)*-Id* and *aph*(*3”*)*-Ib*, and both genes confer resistance to streptomycin (44) (which was, however, not phenotypically tested in these Acinetobacter strains). Both genes were adjacent to each other and are flanked by IS elements, with IS*Aba1* upstream of *aph*(*3”*)*-Ib* and IS*1006* downstream of *aph*(*6*)*-Id* (Fig. 2).

The *sul2* gene that encodes dihydropteroate synthase, which confers sulfonamide resistance, is sandwiched between IS*Aba1* upstream and IS*Cfr1* downstream in both pAC1633-1 and pAC1530 (Fig. 2). In the chromosome of A. baumannii ATCC 19606, *sul2* is associated with the IS*CR2* element and is part of a large (36,157 bp) genomic island designated GI*sul2* (45). The association of *sul2* with the IS*CR2* element was previously reported in the plasmid RSF1010 (46). However, in the GC1 A. baumannii RUH875, IS*Aba1* was detected upstream of *sul2* and provided a promoter for its expression (47). This was observed in pAC1633-1 and pAC1530, except in these two plasmids, the IS*CR2* element was truncated due to the insertion of the 1,617-bp IS*Cfr1*.

**FIG 3:**
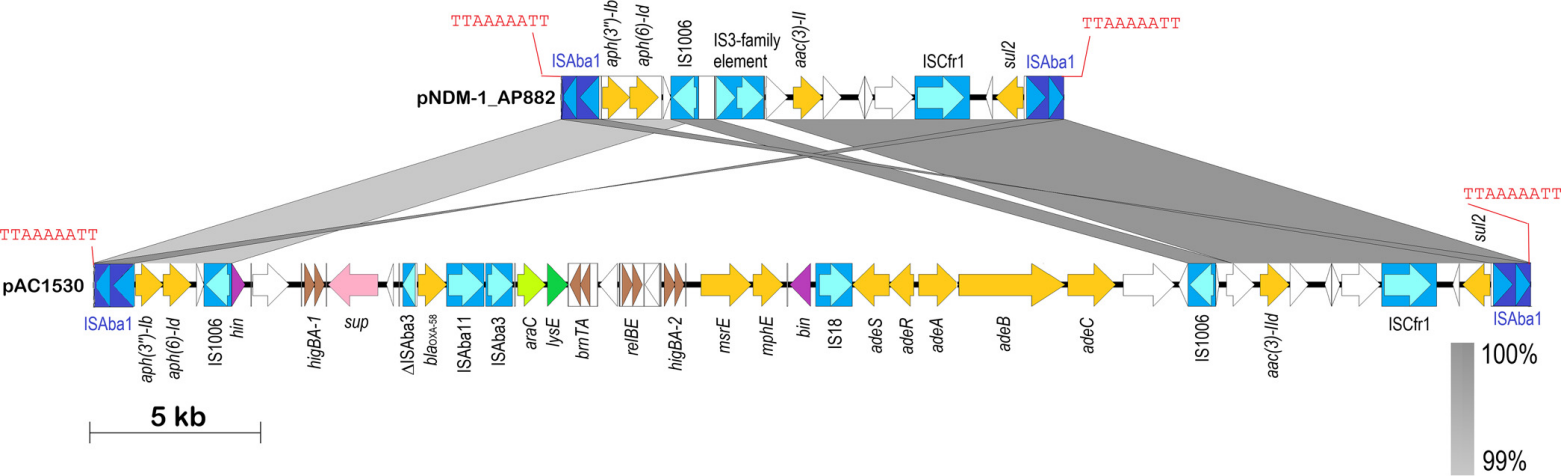
Linear map of the IS*Aba1*-flanked composite transposon Tn*6948* in the A. pittii AP882-derived plasmid pNDM-1_AP882 in comparison with the transposon in pAC1530. Arrows indicate extents and directions of genes and ORFs. Insertion sequence (IS) elements are depicted as blue-colored boxes with lighter blue arrows showing the directions of the transposase genes. IS*Aba1* that flanks the composite transposon is depicted in darker shades of blue. The 9-bp direct repeat sequences that flank the composite transposon are shown in red fonts. Antimicrobial resistance genes are shown as yellow arrows; brown arrows are toxin-antitoxin systems; purple arrows are putative DNA recombination genes; other colored arrows are genes with known homologs; white arrows are ORFs encoding hypothetical proteins. Gray-shaded areas indicate regions with >99% nucleotide sequence identities. The linear map of pNDM-1_AP882 covers nt 146571 to 146597 and continues with nt 1 to 14741 of accession no. CP014478, whereas the map of pAC1530 covers nt 147515 to 173972 and continues with nt 1 to 15685 of accession no. CP045561.1. Note that plasmid pAC1633-1 (accession no. CP059301) is almost identical to pAC1530 within this region except for the omission of the *relBE* toxin-antitoxin system and its downstream ORF, as detailed in the text and in Fig. 2.

The three aminoglycoside resistance genes, *aac*(*3*)*-IId*, *aph*(*6*)*-Id* and *aph*(*3′’*)*-Ib*, along with the *sul2* gene, were found to be in a 42,125-bp fragment in pAC1530 that was flanked by IS*Aba1* with characteristic 9-bp direct repeats of the target sequence in a typical composite transposon-like structure. This 42-kb fragment also included the *bla*_OXA-58_, *msrE*, *mphE*, and *adeRS-adeABC* resistance genes that were nested in a 29,670-bp fragment flanked by IS*1006* (Fig. 3) and which is postulated to be derived from a smaller, separate plasmid via IS*1006*-mediated recombination/transposition. Comparative sequence analysis subsequently indicated that the IS*Aba1*-flanked composite transposon, designated Tn*6948* by the Transposon Registry (48), is 14,750 bp and details of its structure and how the 42-kb region in pAC1530 (as well as pAC1633-1) came about will be presented in a later section of the manuscript.

The *bla*_NDM-1_ gene is found within a 10,099-bp Tn*125* composite transposon that was made up of a pair of flanking IS*Aba125* elements and is a common genetic vehicle for the dissemination of *bla*_NDM_ genes in Acinetobacter spp. (22, 23, 49). One copy of IS*Aba125* is 93 bp upstream of *bla*_NDM-1_ and the presence of an outward-directing promoter at the terminal inverted repeat of IS*Aba125* likely drives the expression of *bla*_NDM-1_ (23, 50). In both pAC1633-1 and pAC1530, Tn*125* was inserted into an unknown open reading frame, resulting in a 3-bp target site duplication (“ACG”), as has been previously reported for this transposon (23, 49, 50), although instances where a 4-bp duplication of the Tn*125* site of insertion have been reported (51).

### The *bla*_OXA-58_ and *msrE-mphE* resistance genes, and the toxin-antitoxin systems, are in p*dif* modules on pAC1633-1 and pAC1530.

**FIG 4:**
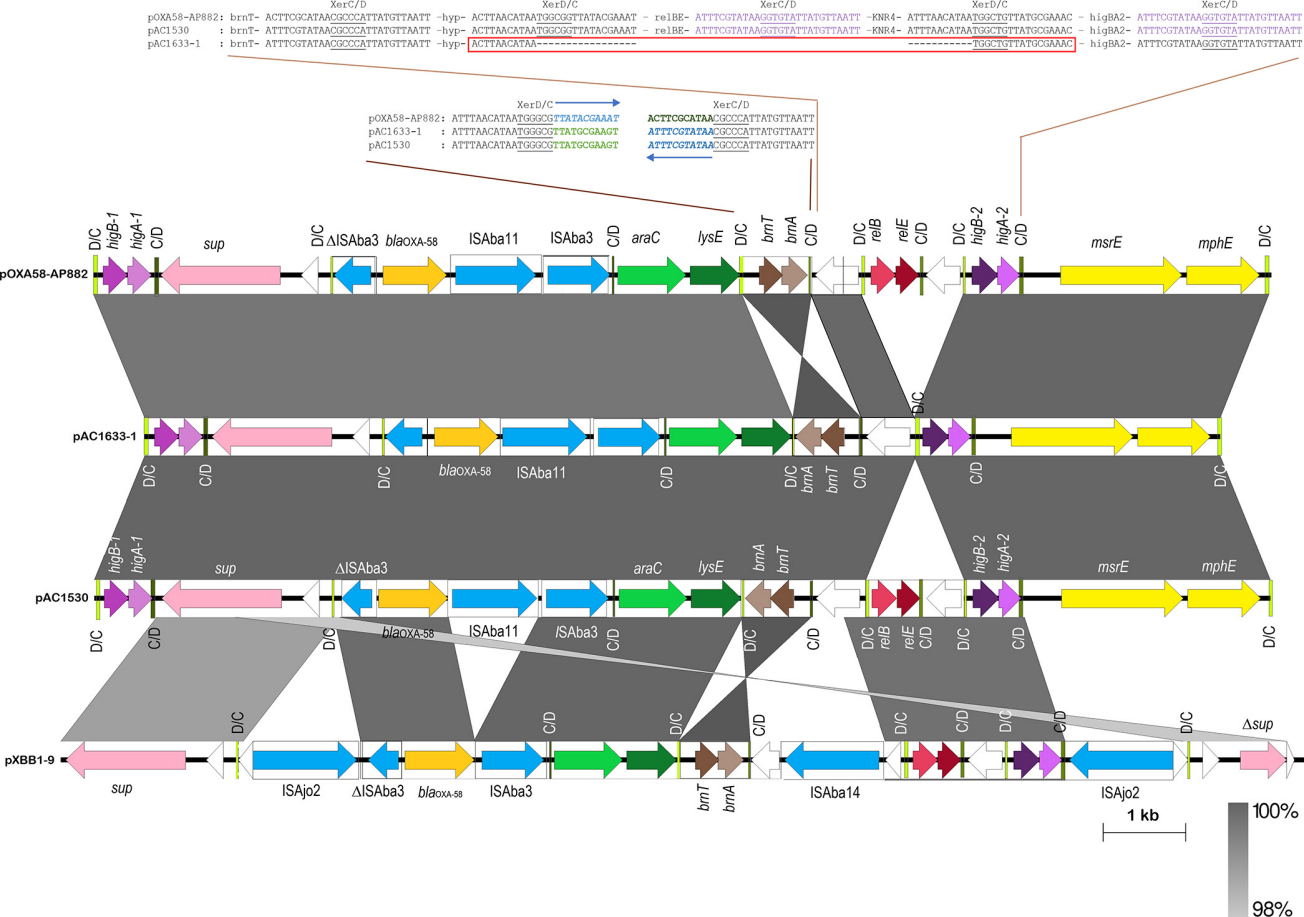
Comparative map of the p*dif*-rich regions surrounding the *bla*_OXA-58_ gene in several Acinetobacter plasmids. Arrows indicate the extents and directions of the genes and ORFs with the *bla*_OXA-58_ gene depicted as a gold arrow, the *msrE* and *mphE* macrolide resistance genes in yellow, and the aminoglycoside resistance gene *aac*(*3*)*-IId* shown in orange. IS elements are shown as boxes with their encoded transposases in blue arrows within the respective boxes. The p*dif* sites are depicted as vertical bars with the orientation of the sites labeled and colored as follows: XerD/XerC colored lime green and labeled as D/C; XerC/XerD colored dark olive green and labeled as C/D. Note the toxin-antitoxin genes that make up the following p*dif* modules: *higBA-1*, *higBA-2*, *brnTA*, and *relBE*. Other genes are labeled as follows: *araC*, putative transcriptional regulator of the AraC family; *lysE*, putative threonine efflux protein; and *sup*, putative sulfate transporter. White arrows depict ORFs that encode hypothetical proteins. The p*dif* sequences (either XerD/C or XerC/D) in pAC1633-1/pAC1530 and pOXA-58_AP882 at the inversion region encompassing *brnTA* and the insertion point of the *relBE* TA system are indicated for the respective plasmids and their relevant regions. For the *brnTA* p*dif* module, note that the XerC sequences in pOXA-58_AP882 are in reverse orientation compared to the corresponding XerC sequences in pAC1633-1 and pAC1530; these are indicated by blue arrows as well as blue and green fonts for the respective p*dif* XerC site. Also note that the p*dif* sequences downstream of the *relE* gene are identical with the p*dif* sequences downstream of the *higA-2* gene, and these sequences are in purple fonts. The p*dif* (XerD/C) site in pAC1633-1 in which the *relBE-*SMI1/KNR4 p*dif* module was inserted in pAC1530 is outlined in a red box and its sequences outlined likewise below. Note that for this particular p*dif* site, the XerD sequences are identical to the XerD sequences of the p*dif* site upstream of *relB* while the 6-bp spacer and XerC sequences are identical to the p*dif* site upstream of *higB-2*. Accession numbers and coverage of plasmid regions for the comparative map are as follows: pOXA-58_AP882 (accession no. CP014479; nt 25121 to 36862 and continued from 1 to 2560); pAC1633-1 (accession no. CP059301; nt 155106 to 168160); pAC1530 (accession no. CP045561.1; nt 153566 to 167840); pXBB1-9 (accession no. CP010351; nt 2158 to 1 and continued from nt 398857 to 398921). The extent of regions with nucleotide sequence identities of between 98 to 100% are shown in gray.

One of the intriguing features of Acinetobacter plasmids is the presence of discrete modules flanked by conserved inverted repeats homologous to the XerC and XerD binding sites (*dif* sites) separated by a 6-bp spacer, which are recombination targets for the XerC and XerD proteins (52–54). Since their initial discovery flanking a discrete module encoding the OXA-24 carbapenemase gene in the A. baumannii pABVA01a plasmid (55), several of these designated p*dif* modules (named for plasmid *dif*) which comprise a pair of inverted p*dif* sites surrounding a gene or several genes, have been described (54, 56). In pAC1633-1 and pAC1530, the region surrounding *bla*_OXA-58_ is rich in p*dif* sites; 11 of the 14 p*dif* sites in pAC1530 were located within a 14,410-bp fragment that spanned nt 153566 to 167912 (Fig. 4). The *bla*_OXA-58_ gene itself is flanked by IS elements (a partial 427-bp IS*Aba3* upstream of *bla*_OXA-58_ and full copies of IS*Aba11* and IS*Aba3* immediately downstream), which are in turn flanked by a pair of inverted p*dif* sites, an arrangement that has been previously reported for the A. johnsonii-encoded plasmid pXBB1-9, but without the presence of IS*Aba11* (57). In pAC1633-1/pAC1530, insertion of IS*Aba11* led to a characteristic 5-bp direct repeat (ATTTA) of the target sequence. In some Acinetobacter, a full IS*Aba3* or IS*Aba3*-like element is found upstream of *bla*_OXA-58_, while in other instances this upstream IS*Aba3* is disrupted by other IS elements (53, 58–60).

A pair of inversely oriented p*dif* sites was also found to flank the *msrE* and *mphE* macrolide resistance genes in pAC1633-1 and pAC1530 (Fig. 4), leading to the formation of a 2,950-bp macrolide-resistance p*dif* module, as had been reported previously in other Acinetobacter plasmids (53). This *msrE-mphE* module has always been found adjacent to a *higBA* TA p*dif* module (53), and this was also the case in pAC1633-1 and pAC1530.

Intriguingly, all known TA systems detected in pAC1633-1 and pAC1530 (*higBA-1*, *brnTA*, and *higBA-2*) were found within this region and they were each flanked by a pair of inverted p*dif* sites. As mentioned earlier, one of the differences between pAC1633-1 and pAC1530 is the addition of a *relBE* TA system and an ORF encoding a protein of the SMI1/KNR4 family in pAC1530, upstream of *higBA-2* (Fig. 4). The *relBE* genes and the genes encoding SMI1/KNR4 protein are also p*dif* modules, as they are each flanked by a pair of inverted p*dif* sites. A closer examination of the p*dif* (XerD/C) sequences showed that in pAC1633-1, the p*dif* (XerD/C) sequences upstream of *higBA-2* are a hybrid of the p*dif* (XerD/C) sequences that flanked the *relBE*-SMI1/KNR4 module in pAC1633-1, i.e., the sequences of the XerD site are identical to the sequences found upstream of *relBE*, while the 6-bp spacer and the XerC site sequences are identical to the sequences upstream of *higBA-2* in pAC1633-1 (Fig. 4). This suggests the *relBE*-SMI1/KNR4 p*dif* module could have been deleted from pAC1530 in pAC1633-1 via a Xer-mediated recombination event.

Another Xer-related rearrangement could be seen when comparing the sequences of pAC1633-1 and pAC1530 with their closest plasmid relative, pOXA-58_AP882, wherein the *brnTA* TA p*dif* module was found to be in inverted orientation. The orientation of *brnTA* in pOXA-58_AP882 is, however, the same in pXBB1-9 (Fig. 4). Interestingly, the XerD and the 6-bp spacer sequences of the flanking p*dif* modules for *brnTA* are identical when comparing pAC1633-1/pAC1530 with pOXA-58_AP882, except the XerC sequences are inverted (pAC1633-1/pAC1530: TTATGCGAAGT; pOXA-58_AP882: ACTTCGCATAA) (Fig. 4). Currently, genome sequencing data strongly support the likelihood that p*dif* modules are mobile, although, to our knowledge, there has yet to be any definite experimental evidence offered or mechanism of mobility elucidated (52). Deletions and inversions of Acinetobacter p*dif* modules have been hinted at (52, 56), and here we show sequence evidence that these do occur.

### pAC1633-1/pAC1530 is likely a hybrid of two plasmids with the cointegration mediated by IS*1006*.

BLASTN analysis of pAC1633-1 and pAC1530 showed they are most similar in sequence to two plasmids found in Acinetobacter pittii AP882 designated pNDM-1_AP882 (accession no. CP014478) and pOXA-58_AP882 (accession no. CP014479). Notably, A. pittii AP882 was isolated from Peninsular Malaysia in 2014 but from a different state (Perak) (61) compared to AC1633-1 and AC1530 (isolated from Terengganu in 2016 and 2015, respectively). When comparing the 146,597-bp pNDM-1_AP882 with pAC1530 and pAC1633-1, pNDM-1_AP882 is nearly identical with pAC1530/pAC1633-1 except for a 1,940-bp region adjacent to IS*1006*. This region contains a 1,472-bp IS-like sequence (nt 4480 to 1472) that encodes two transposases characteristic of the IS*3* family flanked by 21/22-bp imperfect inverted repeats and a 5-bp direct repeat (ACCTG) of the target sequence (Fig. S1). Further analysis of pNDM-1_AP882 led to the discovery of a 14,750-bp composite transposon designated Tn*6948*, formed by flanking IS*Aba1* sequences with a 9-bp target site duplication (TTAAAAATT) that is characteristic of this IS element (Fig. 3). The target site duplication is only found for the entire transposon structure but not for each individual IS*Aba1* element, thus suggesting this is likely an active transposon. Tn*6948* harbors the *sul2* sulfonamide resistance gene and the aminoglycoside resistance genes *aph*(*3”*)*-Ib*, *aph*(*6*)*-Id*, and *aac*(*3*)*-IId*. Tn*6948* has an overall GC content of 50.6% compared to 38 to 39% for the surrounding genes, thus supporting its possible non-Acinetobacter origin.

10.1128/mSphere.01076-20.2 FIG S1 (A) Circular map of plasmid pNDM-1_AP882 from Acinetobacter pittii AP882 (accession no. CP014478). The single IS*1006* copy is indicated in red. The 1,940-bp region adjacent to IS*1006* that differed from pAC1530/pAC1633-1 and contained an IS-like element of the IS*3* family is indicated in a pale yellow box. The two IS*Aba1* elements that flank the composite transposon Tn*6948* are highlighted in blue boxes. Antimicrobial resistance genes, including *bla*_NDM-1_, are shown as gold and orange arrows; other IS elements are shown as gray boxes with their encoded transposases in blue arrows. Green arrows are genes/ORFs that are likely involved in conjugative gene transfer, with darker green arrows showing genes that have homology with known conjugative transfer genes. Sky blue arrows are genes involved in DNA recombination and repair. Maroon arrows are ORFs/CDS encoding hypothetical proteins. (B) Circular map of plasmid pOXA-58_AP882 from Acinetobacter pittii AP882 (accession no. CP014479) showing distribution of p*dif* sites. The two copies of IS*1006* are depicted in red, IS-encoded transposases are shown by blue arrows. The 7,191-bp fragment of pOXA-58_AP882 that is absent from pAC1530 and pAC1633-1 is labeled and highlighted in yellow. This region contains the *mobA/L* gene, the *repB*_GR12_ gene encoding a Rep3 family plasmid replicase of the Acinetobacter GR12 family, an ORF encoding a helix-turn-helix putative DNA-binding protein (depicted as a dark green arrow downstream of *repB*_GR12_), and a putative *relE-xre* toxin-antitoxin system shown as black arrows. Other TA systems are also indicated as black arrows and labeled accordingly. p*dif* sites are depicted as horizontal bars and are labeled as per the orientation of their XerD- and XerC-binding sites (i.e., either XerC/D or XerD/C). The *bla*_OXA-58_ gene is shown as a yellow arrow and labeled. Download FIG S1, TIF file, 2.8 MB. Copyright © 2021 Alattraqchi et al. 2021 Alattraqchi et al. This content is distributed under the terms of the Creative Commons Attribution 4.0 International license.

**FIG 5:**
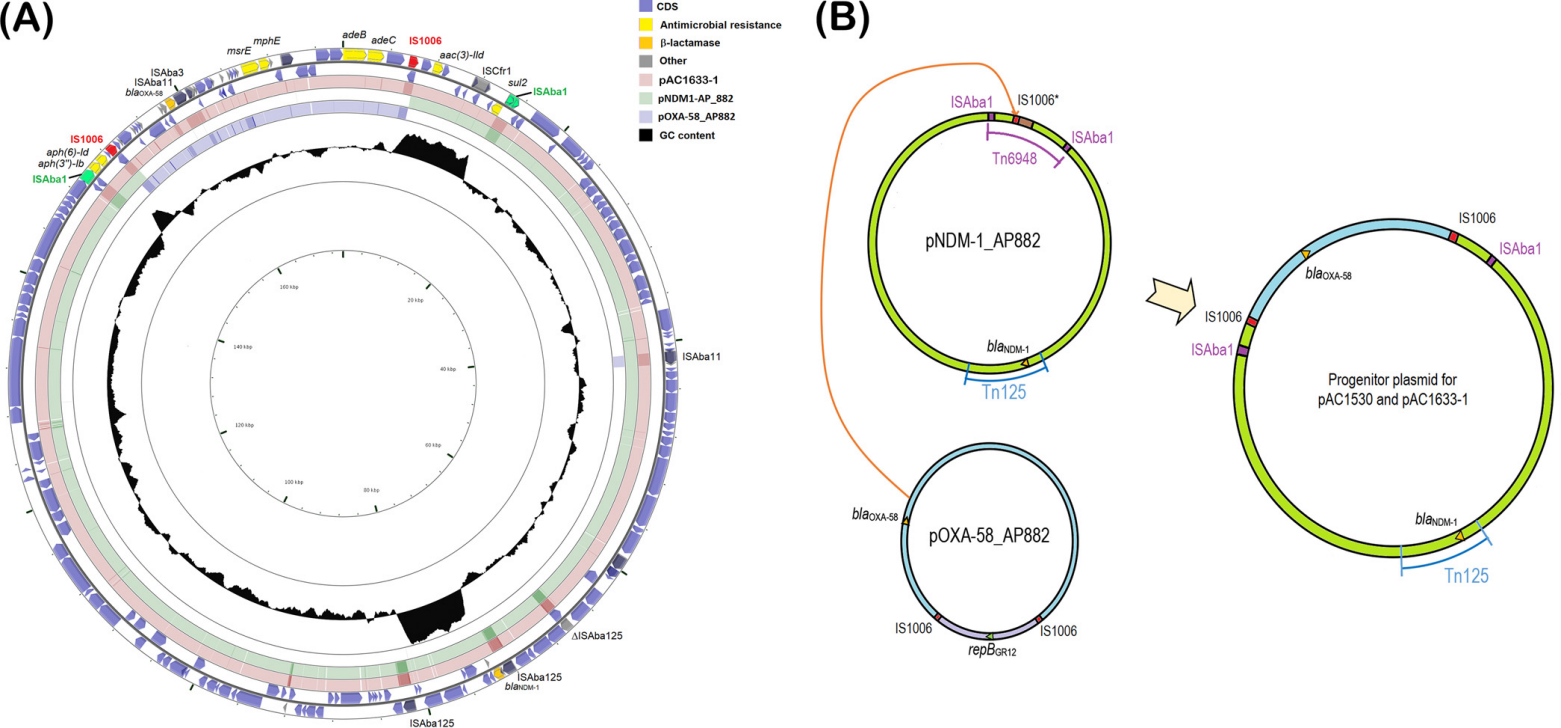
(A). Comparison of pAC1633-1 with plasmids pNDM-1_AP882 and pOXA-58_AP882 from Acinetobacter pittii strain AP882. The outer two circles show the genes and coding sequences (CDS) from pAC1633-1 with the two copies of IS*1006* marked in red and the antimicrobial resistance genes in yellow. The two IS*Aba1* elements that flank the composite transposon Tn*6948* in pNDM-1_AP882 are shown in green. The pink-colored ring indicates pAC1633-1, while the green- and blue-colored inner rings show regions of pNDM-1_AP882 and pOXA-58_AP882, respectively, that share >95% nucleotide sequence identities with the corresponding region in pAC1633-1. The inner black ring depicts a plot of the GC content of pAC16331 with the baseline set as the average GC content of 38% for the plasmid. Note the Tn*125* encoding *bla*_NDM-1_ and Tn*6948* had higher GC content (∼50%) than the average, with their plots extending outward from the circle. Darker shades of pink, green, and blue indicate repeat regions (usually IS elements). Note that pAC1633-1 could possibly came about through integration of the IS*1006*-flanked region that encompasses the *bla*_OXA-58_, *msrE*, and *mphE* resistance genes into the single IS*1006* copy of pNDM-1_AP882, as illustrated in panel B. (B) Hypothetical IS*1006*-mediated cointegration for the generation of the predecessor plasmid for pAC1530 and pAC1633-1 from pNDM-1_AP882 and pOXA-58_AP882. The 29,671-bp region of pOXA-58_AP882 that contains the *bla*_OXA-58_ gene and is flanked by IS*1006* (labeled in light blue) likely recombined with pNDM-1_AP882 at its single IS*1006* region, leading to the formation of the cointegrate predecessor plasmid for pAC1530 and pAC1633-1 that harbored both *bla*_OXA-58_ and *bla*_NDM-1_. The light purple region in pOXA-58_AP882 contains the *repB* replicase gene of the Acinetobacter GR12 family and was not involved in the plasmid cointegrate formation. IS*1006* is depicted as red boxes and labeled. pNDM-1_AP882 harbored an IS*1006* with a T175C mutation (compared with the IS*1006* sequences in pOXA-58_AP882) and is labeled as IS1006*. The brown-colored box in pNDM-1_AP882 refers to a 1,940-bp region adjacent to IS1006* that was absent in pAC1530 and pAC1633-1 and contained an IS*3* family element. This region could have been deleted during the IS*1006*-mediated cointegration process or after the formation of the cointegrate plasmid. The IS*Aba1* elements that flank the composite transposon Tn*6948* in pNDM-1_AP882 are indicated in purple boxes and labeled.

When comparing the 36,862-bp pOXA-58_AP882 plasmid with pAC1530 and pAC1633-1, a 29,671-bp fragment of pOXA-58_AP882 was found to be identical to pAC1530/pAC1633-1, and this region, which comprises the resistance genes *bla*_OXA-58_, *msrE*, *mphE*, and *adeABC-adeRS*, is flanked by two copies of IS*1006* (Fig. 5A). The remaining 7,191 bp of pOXA-58_AP882 that was absent in pAC1530/pAC1633-1 encodes 10 ORFs and this includes ORFs that encode a MobA/L mobilization protein, a Rep3 family Acinetobacter replicase of the GR12 group (62), a hypothetical protein with a helix-turn-helix motif that had previously been misannotated as RepA (43), a protein of the RelE/ParE toxin family and, downstream of it, an ORF that encodes another helix-turn-helix protein but of the Xre family (Fig. S1). The putative RelE/ParE family toxin in this region of pOXA-58_AP882 shared only 27% amino acid sequence identity with the RelE toxin of the previous RelBE TA pair found within this plasmid as well as in pAC1530 (but which was absent in pAC1633-1). In comparing the IS*1006* sequences in these plasmids, it was found that the two IS*1006* copies in pOXA-58_AP882 were identical with the two copies in pAC1530 and pAC1633-1. However, the solitary IS*1006* copy in pNDM-1_AP882 had a single nucleotide change in which T replaced C at nt 175 of the 819-bp IS*1006*.

It is thus tempting to speculate that the 29,671-bp region of pOXA-58_AP882 that contained *bla*_OXA-58_ formed a composite transposon-like structure flanked by two copies of IS*1006* and that this region could have transposed or recombined with pNDM-1_AP882 at its single IS*1006* copy residing within Tn*6948*. This transposition or recombination might have resulted in a predecessor for plasmids pAC1530 and pAC1633-1, which contain both the *bla*_NDM-1_ and *bla*_OXA-58_ genes in a single plasmid that has two copies of IS*1006* (Fig. 5B). IS*1006* belongs to the large IS*6*/IS*26* family of IS elements (63) and this family, in particular IS*26*, has been known to mediate the formation of cointegrates between two DNA molecules, with the donor molecule harboring IS*26* (64). However, this route, designated “replicative” or “copy-in,” usually leads to the formation of an 8-bp target site duplication for the IS*26* and inserts at random sites (63). Here, no target site duplication could be detected in pAC1530, pAC1633-1, or even pOXA-58_AP882 at the ends of the IS*1006*-flanked region. Nevertheless, IS*26* was recently demonstrated to perform a unique transposase-dependent reaction when both donor and target molecules carry a copy of IS*26*. This reaction, designated “targeted conservative,” is targeted, occurring at one or the other end of the two IS*26* elements, and with the IS element not duplicated and a target site duplication not generated (65). Cointegration by the targeted conservative route was found to be the preferred reaction if two copies of IS*26* in two different DNA molecules are available (65, 66). Based on sequence analysis alone, it is difficult to ascertain the mechanism by which the predecessor cointegrate plasmid for pAC1530 and pAC1633-1 was formed, i.e., whether it was through the targeted conservative route since both pOXA-58_AP882 and pNDM-1_AP882 harbored IS*1006*, or by homologous recombination via IS*1006*, or even by classical transposition—as the two copies of IS*1006* that flanked the 29,671-bp *bla*_OXA-58_ fragment do form a composite transposon structure, albeit without the characteristic target site duplications at its termini.

### Transmissibility of pAC1530 and pAC1633-1.

**FIG 6:**
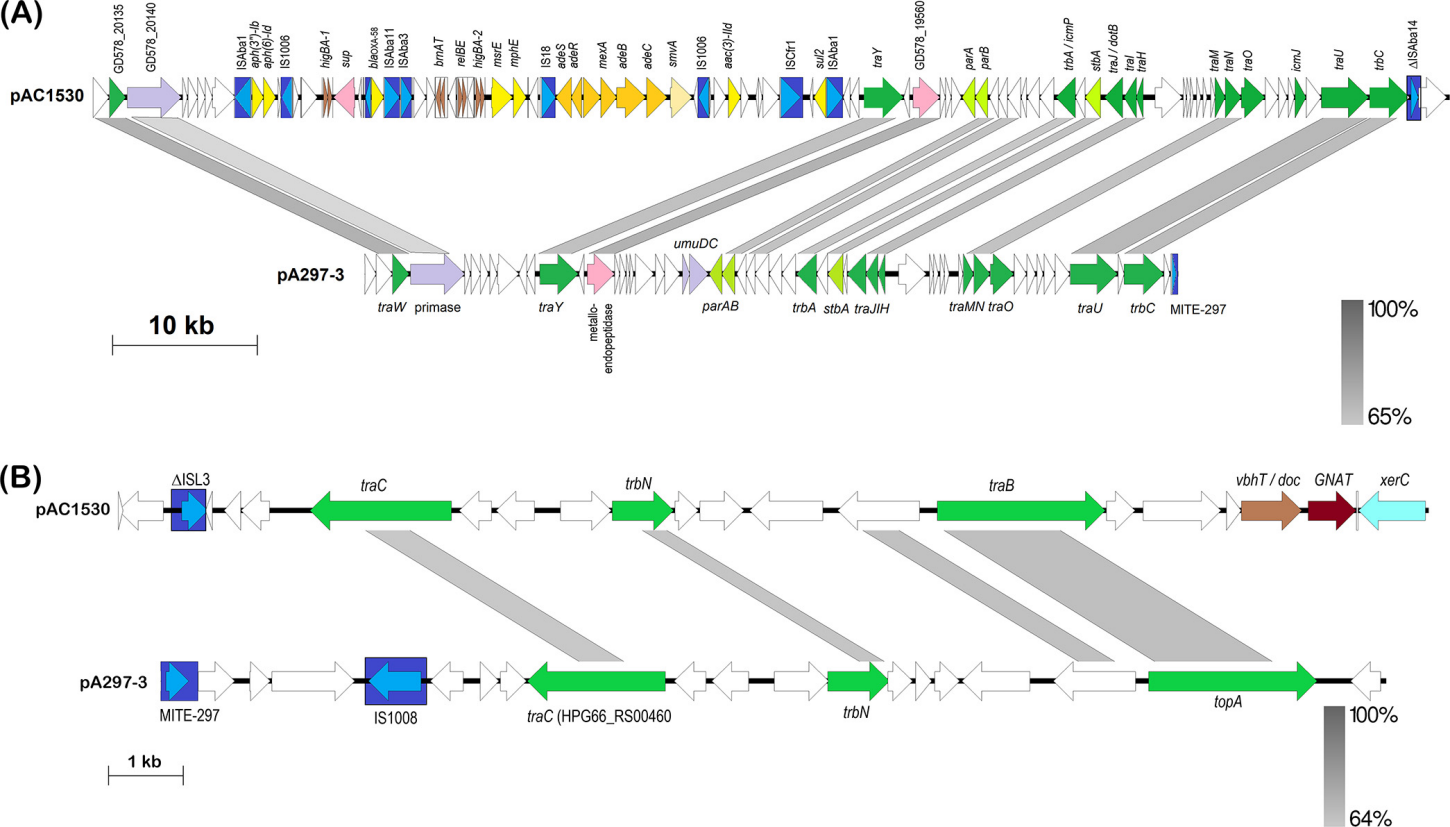
Linearized map of the two conjugative regions of pAC1530 compared to pA297-3. (A) Region 1; map shows nt 137720 to 173972 and continues with 1 to 57600 of pAC1530 (numbered as in accession no. CP045561.1) and the reverse complement of nt 169947 to 200633, continues with 1 to 25440 of pA297-3 (numbered as in accession no. KU744946.1). (B) Region 2; map depicts nt 80481 to 98080 of pAC1530 and the reverse complement of nt 76001 to 92455 of pA297-3. Arrows indicate the extents and directions of genes and ORFs with identified conjugative transfer genes depicted as dark green arrows. Lime green arrows are the plasmid partitioning genes *parA* and *parB*. IS elements and the miniature inverted-repeat transposable element (MITE) identified in pA297-3 (67) are depicted as dark blue boxes with their encoded transposase shown as lighter blue arrows. Antibiotic resistance genes in pAC1530 are indicated as yellow arrows while toxin-antitoxin genes are shown as brown arrows. Other identified genes are in purple and pink arrows, with white arrows indicating ORFs encoding hypothetical proteins. The IS*Aba1*-flanked Tn*6948* is indicated; note that in pAC1530 and pAC1633-1, Tn*6948* is interrupted by the IS*1006*-flanked region that contains antimicrobial resistance genes such as *bla*_OXA-58_, *sul2*, *msrE*, and *mphE* (see Fig. 3 and text). Gray-shaded areas indicate regions with DNA sequence identities as indicated by the bars at the bottom right of each figure. Note that although the figure depicts only pAC1530, pAC1633-1 is nearly identical to pAC1530 in Region 1 except for an insertion of IS*Aba11* in the ORF upstream of *traM* and deletion of the *relBE* p*dif* module (see Fig. 2 and text).

The fact that pAC1530 and pAC1633-1 were nearly identical, large (>170 kb) plasmids that were isolated from two different Acinetobacter species in two different years (A. nosocomialis AC1530 from 2015 and A. baumannii AC1633 from 2016) but from the same hospital is suggestive of plasmid transmissibility. Sequence analysis also indicated the presence of several conjugative transfer-related genes, most of which share between 50 to 70% amino acid sequence identities with the corresponding translated proteins of the conjugative plasmid pA297-3 from A. baumannii isolate A297 (67) (Table 4). The conjugative transfer genes of pAC1530 and pAC1633-1 were broadly distributed in two large regions of the plasmids, as they were in pA297-3. The order of the transfer genes in both regions in pAC1530 and pAC1633-1 (designated regions 1 and 2) was identical with the order in pA297-3, despite that the nucleotide sequence identities were lower than 65% in some parts of these two regions (Fig. 6). However, in both pAC1530 and pAC1633-1, region 1, which spans from *traW* to *trbC*, was interrupted by a 42-kb fragment encompassing the IS*Aba1*-flanked composite transposon Tn*6948* and, nested within it, the 29-kb IS*1006*-flanked fragment derived from pOXA-58_AP882 (Fig. 6). Conjugation assays were performed using the carbapenem resistant parental hosts, A. baumannii AC1633 and A. nosocomialis AC1530, as donor strains and, as recipients, carbapenem susceptible A. baumannii ATCC 19606 and A. baumannii AC1529 clinical isolate that were induced to sodium azide resistance with MIC values of >300 μg/ml. Despite repeated attempts with established conjugation assay protocols (67, 68) and using different ratios of donor to recipient cells, no transconjugants were obtained that were able to grow on the selection plates (LB agar supplemented with 10 μg/ml imipenem and 300 μg/ml sodium azide). Thus, we were unable to provide direct laboratory experimental evidence that pAC1530 and pAC1633-1 were transmissible. The 200-kb pA297-3 plasmid from A. baumannii A297, which encodes the *sul2* sulfonamide and *strAB* streptomycin resistance genes, was found to transfer sulfonamide and streptomycin resistance to a rifampin-resistant A. baumannii ATCC 17974 strain at a high frequency of 7.20 × 10^−2^ transconjugants/donor (67). However, two other plasmids, pD4 and pD46-4, which shared the transfer regions with pA297-3, were found to be nontransmissible (69, 70). In the case of pD4, an IS*Aba25*-like element was inserted into the DNA primase gene downstream of *traW*, indicating the possibility that this gene could be involved in conjugative transfer (69). However, for pD46-4, no such IS or other genetic elements were found to have interrupted the conjugative transfer-related genes. Also, no SNPs were detected in the transfer genes that might have led to a frameshift or a premature stop codon within these genes (70). The reason for the apparent nontransmissibility of pD46-4 compared to pA297-3 was not known (70). As for pAC1530 and pAC1633-1, their apparent nontransmissibility could be attributed to the insertion of the 42-kb fragment containing Tn*6948* and the IS*1006*-flanked resistance region from pOXA-58_AP882 into the conjugative transfer region 1. However, the insertion of Tn*6948* at the same site was already present in pNDM-1_AP882, although, here the insertion was only 14.2 kb (Fig. 3). There was also no experimental evidence of the transmissibility of pNDM-1_AP882, although in this case the recipient strain used was an azide-resistant derivative of E. coli J53 (61). Nevertheless, the genomic sequence evidence presented here strongly infers the transmissible nature of pNDM-1_AP882 and, by extension, pAC1530 and pAC1633-1, as these three highly related plasmids were found in three different species of Acinetobacter. Perhaps the rate of conjugative transfer for these plasmids was exceptionally low and thus below detectable limits, in stark contrast to what was reported for pA297-3 in which the conjugative transfer region 1 was uninterrupted. Alternatively, successful conjugative transfer of these plasmids may require certain environmental or media conditions that were not met when the experiments were conducted in the laboratory using established protocols. Further work is clearly needed to resolve this transmissibility conundrum for pAC1530 and pAC1633-1.

**TABLE 4:** Conjugative transfer-related genes identified in pAC1530 and pAC1633-1 compared to their corresponding genes in pA297-3

Gene	Product size pAC1530^*a*^ (aa)	Product size pA297-3^*b*^ (aa)	% Protein identity^*d*^	Possible function^*c*^
Region 1				
*traW*	378	377	75 (282/376)	Lipoprotein
*traY*	902	882	66 (526/729)	Integral membrane protein
*trbA*	445	456	62 (273/443)	Formation of thick pilus/DNA transfer
*traJ*	408	429	62 (262/410)	DNA-binding protein
*traI*	277	272	69 (192/279)	Lipoprotein
*traH*	156	156	66 (105/158)	Lipoprotein
*traM*	232	238	60 (133/231)	Formation of thick pilus/DNA transfer
*traN*	367	376	63 (237/379)	Signal peptide
*traO*	541	543	50 (252/501)	Formation of thick pilus/DNA transfer
*icmJ*^*e*^	255	252	60 (149/250)	
*traU*	1088	1090	70 (754/1084)	Nucleotide-binding protein
*trbC*	913	912	61 (569/912)	Nucleotide-binding protein
Region 2				
*traC*	632	616	49 (305/619)	Inner membrane complex ATPase
*trbN*^*f*^	272	275	50 (142/266)	Major pilus subunit
*traB*	750	753	60 (451/753)	Outer membrane complex

a GenBank accession no. CP045561.1.

b GenBank accession no. KU744946.1 (Hamidian et al., 2016) (67).

c Predicted function as listed in Hamidian et al. (2016) (67) and Christie (2016) (91).

d Numbers in parentheses indicate amino acid identities.

e *icmJ* as annotated by PROKKA; shared 60% amino acid sequence identity with HPG66_RS00900 of pA297-3, which was annotated as hypothetical; low sequence identities (42%; 18/43) with IcmJ of Legionella pneumophila (accession no. CAH11669.1).

f *trbN* was annotated by Hamidian et al. (2016) (67) for pA297-3 and pD46-4 (Nigro and Hall, 2017) (70); no detectable similarities with other conjugal proteins.

### The *tetA*(*39*) tetracycline resistance gene in pAC1633-2 is within a p*dif* module.

**FIG 7:**
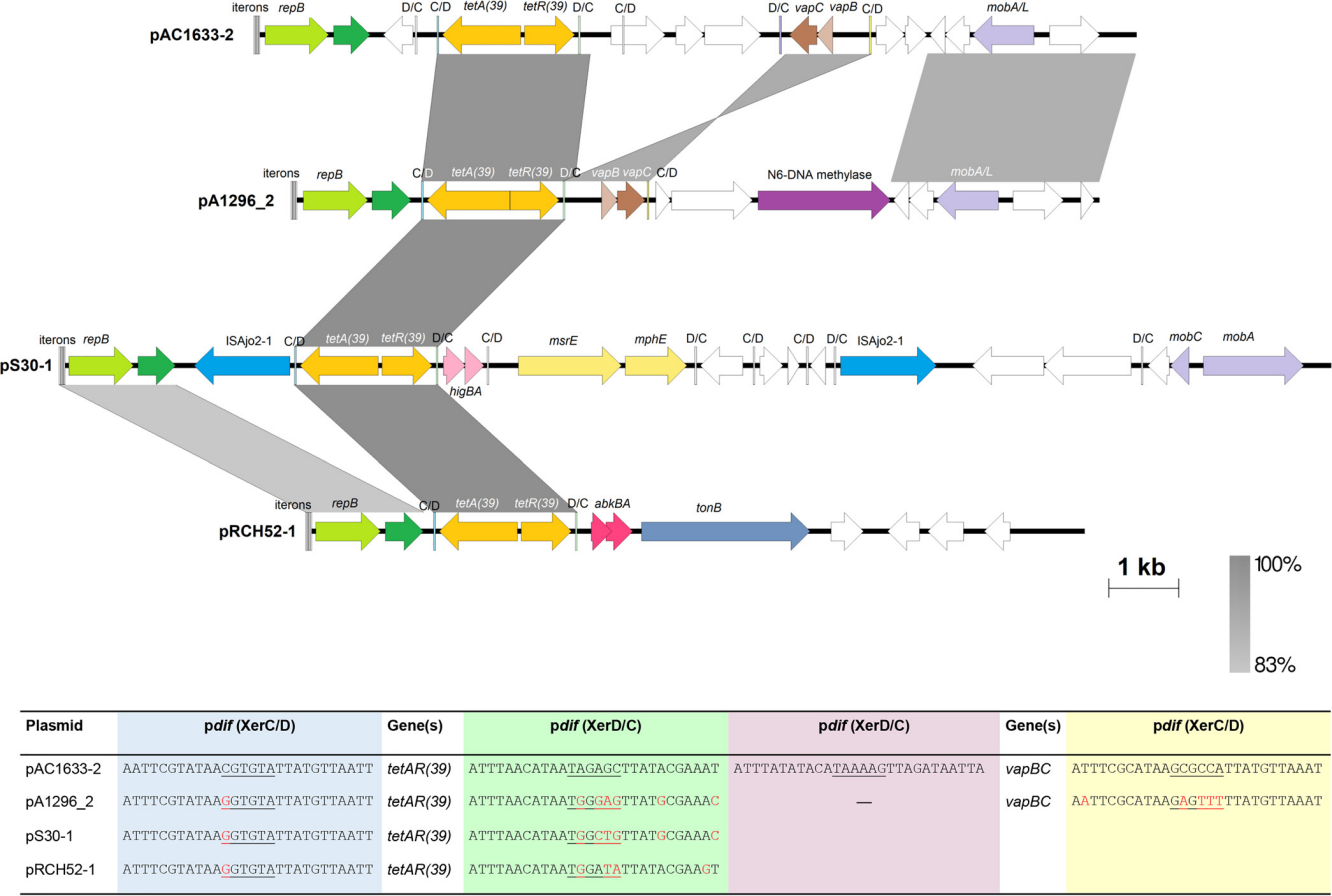
Linearized map of pAC1633-2 compared to other similar plasmids harboring the *tetAR*(*39*) p*dif* module. Arrows indicate the extents and directions of genes and ORFs with yellow arrows for the *tetA*(*39*) and *tetR*(*39*) tetracycline resistance genes and pale yellow arrows for the macrolide resistance genes *msrE* and *mphE* in pS30-1. Plasmid replicase genes of the Rep3 family are depicted as green arrows and labeled *repB*, while darker green arrows are ORFs encoding putative DNA-binding proteins that have been previously misannotated as *repA* (43). Mobilization-related genes are shown as light purple arrows and the gene encoding an N6-DNA methylase is in dark purple. The *vapBC* toxin-antitoxin genes in pAC1633-2 and pA1296_2 are indicated as brown arrows; *higBA* in pS30-1 are depicted in pink; whereas *abkBA* in pRCH52-1 are in dark pink. The transposase encoded by ISAjo2 in pS30-1 is colored blue. White arrows are ORFs that encode hypothetical proteins. Regions with significant DNA identities from 83 to 100% are shaded in shades of gray as indicated in the bar at the bottom right of the figure. The four 22-bp iterons that are the likely *oriV* site for each plasmid is shown as successive horizontal bars at the beginning of the plasmid linear maps. The p*dif* sites are depicted as horizontal bars labeled as C/D for the XerC-XerD orientation and D/C for XerD-XerC orientation. Note that the orientation of the *vapBC* genes flanked by p*dif* sites in pAC1633-2 are inverted compared to pA1296_2. The XerC/D and XerD/C sites flanking the *tetAR*(*39*) genes are colored light blue and light green, respectively, whereas the XerC/D and XerD/C sites flanking the *vapBC* genes in pAC1633-2 are colored light yellow and light purple, respectively. The sequences for the p*dif* sites flanking the *tetAR*(*39*) genes as well as the *vapBC* TA genes are shown in the table at the bottom of the figure. Bases highlighted in red are those that differ from the p*dif* sequences of pAC1633-2. The accession numbers of the plasmids used in this analysis are as follows: pA1296_2 (accession no. CP018334), pS30-1 (accession no. KY617771), and pRCH52-1 (accession no. KT346360).

Plasmid pAC1633-2 is 12,651 bp and encodes a Rep3 family replicase of the Acinetobacter GR8/GR23 group that was preceded by four 22-bp iterons characteristic of Rep3 family plasmids (42, 43). pAC1633-2 also encodes a *tetA*(*39*) tetracycline resistance gene which was adjacent to and divergently transcribed from a *tetR*(*39*) regulatory gene (Fig. 7). This 2,001-bp fragment is identical with the *tetAR*(*39*) genes that made up a p*dif* module in plasmids pS30-1, pRCH52-1, and pAB1-H8 (53). However, the p*dif* sites that flank this *tetAR*(*39*) region in pAC1633-2 differed from those in pS30-1, pRCH52-1, and pAB1-H8 at the 6-bp spacer and the XerC-recognition site (Fig. 7). Another p*dif* module that was detected in pAC1633-2 encodes the *vapBC* toxin-antitoxin system, which was in an inverted orientation compared with plasmid pA1296_2 from A. baumannii A1296 (accession no. CP018334) (Fig. 7).

AC1633-2 also harbors a *mobA/L* gene that encodes a relaxase of the MOB_Q_ family, indicating the possibility of the plasmid being mobilized should a suitable conjugative plasmid be present in the host cell. Since AC1633 also harbored the potentially conjugative pAC1633-1 plasmid, the ability of pAC1633-1 to mobilize pAC1633-2 was tested in conjugation experiments by selecting for transconjugants that exhibit tetracycline resistance in addition to azide resistance. Despite repeated experiments, no such transconjugants were detected, indicating that pAC1633-1 was unable to mobilize pAC1633-2. This, however, does not rule out the possibility that pAC1633-2 could be mobilized by a different type of conjugative plasmid to pAC1633-1.

### Two other plasmids in A. baumannii AC1633, pAC1633-3 and pAC1633-4, are cryptic.

Two other smaller plasmids are found in A. baumannii AC1633, the 9,950-bp pAC1633-3 and the 5,210-bp pAC1633-4. Neither plasmid carries any antimicrobial or metal resistance genes, nor any genes that could confer a specific phenotype to their host, and are thus characterized as cryptic plasmids. Both plasmids encode replicases of the Rep3 superfamily that are common in Acinetobacter plasmids, with pAC1633-3 belonging to the GR28 group where pAC1633-4 belongs to the GR7 group (42, 43). pAC1633-3 and pAC1633-4 could potentially be mobilizable, as they carry *mobA/L* genes of the MOB_Q_ family and pAC1633-4 also carries a *mobS*-like gene. However, in the absence of any selectable marker, we were unable to determine if these two plasmids could be mobilized by pAC1633-1.

Four p*dif* sites were detected in pAC1633-3 but none in pAC1633-4. Interestingly, one of the p*dif* modules in pAC1633-3 is 464 bp, encodes a putative protein of the SMI1/KNR4 family, and is identical with the p*dif* module that was found downstream of the *relBE* p*dif* module in the A. nosocomialis AC1530-derived pAC1530. This SMI1/KNR4 p*dif* module, along with the *relBE* p*dif* module, is absent in pAC1633-1 and is one of the features that differentiates pAC1530 from pAC1633-1. The other p*dif* module in pAC1633-3 is 4,331 bp and encodes a putative regulatory protein of the Xre family, a *hipA*-like toxin, and a 602-amino acid protein of the DEAD/DEAH box family of helicases.

In summary, complete genome sequencing of carbapenem-resistant A. baumannii AC1633 and A. nosocomialis AC1530 isolates led to the discovery of a ca. 170-kb plasmid that encodes the NDM-1 and OXA-58 carbapenemases along with several other resistance determinants and was likely responsible for the MDR status of these two clinical isolates. The A. baumannii AC1633-derived pAC1633-1 and the A. nosocomialis AC1530-derived pAC1530 were nearly identical except for the insertion and deletion of IS elements and a p*dif* module. Both plasmids were a patchwork of multiple mobile genetic elements, with the *bla*_NDM-1_ gene residing in a Tn*125* composite transposon while *bla*_OXA-58_ was flanked by IS elements nested within a p*dif* module. The *msrE-mphE* macrolide resistance genes were also located within a p*dif* module, as were several toxin-antitoxin genes, highlighting the importance of these Xer recombination-dependent modules as one of the drivers of plasmid diversity in Acinetobacter. Comparative sequence analysis indicated that pAC1633-1/pAC1530 is likely a cointegrate of two plasmids which separately encode the *bla*_NDM-1_ and *bla*_OXA-58_ genes in an A. pittii clinical isolate, and that was formed via an IS*1006*-mediated recombination or transposition event. Horizontal transmission of pAC1633-1/pAC1530 was inferred from the discovery of the almost identical plasmid in two different species of Acinetobacter from the same hospital, though this could not be experimentally replicated in the laboratory. Nevertheless, the presence of such large, potentially transmissible multidrug resistance plasmids in Acinetobacter that coharbor the NDM-1 and OXA-58 carbapenemases in this and other recent reports (51, 57) warrants monitoring and assessment of the risk of spread of these plasmids to susceptible strains, particularly in healthcare settings.

## MATERIALS AND METHODS

### Ethical approval, bacterial isolates, and antimicrobial susceptibility profiles.

Ethical approval for this study was obtained from the Malaysian Ministry of Health’s National Medical Research Register (approval no. NMRR-14-1650-23625-IIR). A. baumannii AC1633 and A. nosocomialis AC1530 were isolated from Hospital Sultanah Nur Zahirah, Kuala Terengganu, Malaysia in 2016 and 2015, respectively. Species identification of both isolates was performed by sequencing of the *rpoB* gene as previously described (13, 71). Antimicrobial susceptibility profiles of both isolates were determined using a panel of 22 antibiotics recommended for Acinetobacter spp. (26) and by disk diffusion (Oxoid Ltd., Basingstoke, UK) on Mueller-Hinton (MH) agar, except for colistin and polymyxin B, which were determined by obtaining the MIC values by the agar diffusion method (25). Carbapenem resistance was validated by determining the MIC values for imipenem, meropenem and doripenem using M.I.C. Evaluator strips (Oxoid Ltd., Basingstoke, UK). Results were interpreted according to the Clinical and Laboratory Standards Institute (CLSI) guidelines (72). Production of metalo-β-lactamases was determined using the Etest MBL kit (bioMérieux, La Balme-les-Grottes, France).

### DNA isolation, whole-genome sequencing, and sequence analyses.

Genomic DNA for whole-genome sequencing was prepared using the Geneaid Presto mini gDNA bacteria kit (Geneaid, Taipeh, Taiwan) following the manufacturer’s recommended protocol and the extracted DNA quality was evaluated using a Qubit 2.0 fluorometer (Life Technologies, Carlsbad, CA). Genome sequencing was performed on the Illumina NextSeq (Illumina Inc., San Diego, CA) and PacBio RSII (PacBio, Menlo Park, CA) platforms by a commercial service provider (Novogene, Beijing, China) and hybrid assembly was carried out using SPAdes (version 3.11.1) (73). Gene prediction for the assembled genomes was performed with PROKKA (74) with annotation achieved using the NCBI Prokaryotic Genome Annotation Pipeline (PGAP) (75). Multilocus sequence typing (MLST) in the Institut Pasteur (76) and Oxford (77) schemes was performed via the A. baumannii MLST database (https://pubmlst.org/abaumannii/) (33). Pan genome analysis for A. baumannii AC1633, A. nosocomialis AC1530, and related global A. baumannii and A. nosocomialis isolates (as listed in Table S1) was determined using ROARY, with the core genomes identified using the criteria of amino acid sequence identities of >95% (78) and presence in 99% of genomes. The derived core genome alignments for A. baumannii and A. nosocomialis were then used to infer maximum-likelihood (ML) trees using FastTree (79) with 100 bootstraps under the GTR time-reversible model. The resulting A. baumannii and A. nosocomialis phylogenetic trees were then visualized using iTOL v5 (https://itol.embl.de/) (80). The A. baumannii and A. nosocomialis genomes chosen for analyses were reference genomes that were either complete and/or have been published. For A. baumannii, unpublished genomes of isolates originating from Malaysia were also included in the analyses.

Antibiotic resistance genes were identified using ResFinder (https://cge.cbs.dtu.dk/services/ResFinder/) (81) and the Comprehensive Antibiotic Resistance Database (CARD) (https://card.mcmaster.ca/) (82), whereas ISFinder (https://isfinder.biotoul.fr/) (41) was used to identify insertion sequences. Toxin-antitoxin systems were identified using the toxin-antitoxin database TADB 2.0 (https://bioinfo-mml.sjtu.edu.cn/TADB2/index.php) (83), and putative conjugative transfer genes were identified using SeCreT4 (https://db-mml.sjtu.edu.cn/SecReT4/) (84). All plasmid sequences were manually inspected using BLAST (https://blast.ncbi.nlm.nih.gov/Blast.cgi) and ORF Finder (https://www.ncbi.nlm.nih.gov/orffinder/) to validate the genes/open reading frames (ORFs) that were predicted by the annotation and other programs. Pfam searches (85) and the NCBI Conserved Domain Database (CDD) (86) were also used to identify possible protein functions. Plasmids similar to pAC1530 and pAC1633-1 were searched by BLASTN (at https://blast.ncbi.nlm.nih.gov/Blast.cgi) using the nonredundant (nr) nucleotide collection and initially optimized for highly similar sequences (MegaBLAST). Regions of high sequence identities with multiple hits (such as the *bla*_NDM-1_-encoded Tn*125*) were then subsequently omitted from subsequent searches, which were optimized for more dissimilar sequences (discontiguous MegaBLAST) and somewhat similar sequences (BLASTN). The presence of p*dif* sites in pAC1530, pAC1633-1, and related plasmids was determined by BLASTN screening using known XerC/XerD and XerD/XerC sites in published reports (52–54) and manually examining hits that were 75 to 80% identical in sequence (54).

SnapGene 5.1.5 (GSL Biotech LLC., San Diego, CA) was used to visualize and manipulate the sequences studied. Figures were drawn to scale using EasyFig 2.2.3 (http://mjsull.github.io/Easyfig/) (87) and CGView (http://stothard.afns.ualberta.ca/cgview_server/) (88).

### Conjugation assays.

Conjugation assays were carried out to investigate the transmissibility of pAC1530 and pAC1633-1 from their respective carbapenem-resistant natural hosts, A. nosocomialis AC1530 and A. baumannii AC1633, to the appropriate susceptible isolates, A. baumannii ATCC 19606 and A. baumannii AC1529. A. baumannii ATCC 19606 is the type strain of A. baumannii that has been widely used in various studies, is resistant to sulfonamides due to the presence of the *sul2* gene in its chromosome, but remains susceptible to a wide range of other antibiotics (45, 89), including the carbapenems. A. baumannii AC1529 was isolated from the blood of a 59-year-old male patient in the emergency ward of Hospital Sultanah Nur Zahirah in 2015 and its identity was confirmed by *rpoB* sequencing (71). AC1529 showed intermediate resistance to cefotaxime and ceftriaxone but was susceptible to the other 20 antimicrobials that were tested, including carbapenems. A. baumannii ATCC 19606 and A. baumannii AC1529 were selected for induction to azide resistance to be used as recipient strains in the conjugation assays. Spontaneous mutation of both A. baumannii strains to sodium azide resistance was performed by continuous exposure to increasing concentrations of sodium azide, as described by Leungtongkam et al. (90).

A. baumannii ATCC 19606 and A. baumannii AC1529 isolates with sodium azide MIC values of >300 μg/ml were used as recipients, whereas A. baumannii AC1633 and A. nosocomialis AC1530 were used as donors in four separate conjugation experiments. Equal amounts of overnight cultures of the donor and recipient cells were mixed and incubated at 37°C on Luria-Bertani (LB) agar plates overnight. Cells were resuspended and diluted in 0.9% NaCl and selected on LB agar plates supplemented with 300 μg/ml sodium azide and 10 μg/ml imipenem. Conjugation assays were also repeated with different ratios of donor to recipient cells (1:2, 1:3, 2:1, and 3:1). To investigate if the *tetA*(*39*)-harboring plasmid pAC1633-2 could be mobilized by pAC1633-1 in A. baumannii AC1633, conjugation experiments involving AC1633 as donor were also plated on LB agar plates supplemented with 300 μg/ml sodium azide and 4 μg/ml tetracycline.

### Data availability.

The complete sequence of the A. baumannii AC1633 chromosome was deposited in GenBank under accession no. CP059300, whereas its four plasmids were deposited under the following accession numbers: pAC1633-1 (CP059301), pAC1633-2 (CP059303), pAC1633-3 (CP059304), and pAC1633-4 (CP059302). The A. nosocomialis AC1530 chromosomal sequence was deposited under accession no. CP045560.1, whereas its plasmid pAC1530 was deposited under accession no. CP045561.1.
