# Supplementary material for: Complete Genome Sequencing of Acinetobacter baumannii AC1633 and Acinetobacter nosocomialis AC1530 Unveils a Large Multidrug-Resistant Plasmid Encoding the NDM-1 and OXA-58 Carbapenemases
Source: mSphere. 2021 Jan 27;6(1):e01076-20. doi: 10.1128/mSphere.01076-20 (PMC7885321; doi:10.1128/mSphere.01076-20)
Supplement: TABLE S1 [file mSphere.01076-20-st001.docx]

**Suppl. Table S1(A)**

| *A. baumannii* Isolate | Source of isolate/ Location/ Year*^a^* | MLST | | Clonal Group | Accession nos. / Reference(s) |
| --- | --- | --- | --- | --- | --- |
|  |  | Oxford | Pasteur |  |  |
| A85 | Sputum / Australia / 2003 | 781^Δ^ | 1 | GC1 | CP021782; (1, 2) |
| A388 | ND / Greece / 2002 | 439 | 1 | GC1 | CP024418.1; (3) |
| AB0057 | Blood /Walter Reed Army Hospital, Washington / 2004 | 207 | 1 | GC1 | CP001182; (4) |
| AB307-0294 | Blood / New York / 1994 | 231 | 1 | GC1 | CP001172.2; (2) |
| AC12 | Blood / Kuala Terengganu / 2011 | 195 | 2 | GC2 | CP007549; (5) |
| AC29 | Blood / Kuala Terengganu / 2011 | 195 | 2 | GC2 | CP007535; (6) |
| AC30 | Blood / Kuala Terengganu / 2011 | 195 | 2 | GC2 | CP007577; (6) |
| ACICU | CSF / Rome / 2005 | 437 | 2 | GC2 | CP031380; (2, 7) |
| ATCC17978 | Blood / ND / 1951 | 112 | 437 |  | CP012004.1; (8, 9) |
| ATCC19606 | Urine / USA / 1948 (type strain) | 931 | 52 |  | CP046654.1; (10) |
| AYE | ND / France / 2001 | 231, 1604^#^ | 1 | GC1 | CU459141.1; (11, 12) |
| BJAB0715 | CSF / Beijing / 2007 | 642 | 23 |  | CP003847.1; (13) |
| BJAB0868 | Ascites / Beijing / 2008 | 218* | 2 | GC2 | CP003849.1; (13) |
| BJAB07104 | Blood / Beijing / 2007 | 368, 1962* | 2 | GC2 | CP003846,1; (13) |
| Canada-BC-5 | ND / Canada / 2006 | 947^§^ | 1 | GC1 | AFDN00000000; (14) |
| CIP70.10 | Skin tissue/ France/1970 | 819 | 126 |  | LN865143; (15) |
| D1279779 | Blood / Darwin, Australia / 2009 | 942 | 267 |  | CP003967.2; (16) |
| IS-123 | Wound / Baghdad, Iraq / 2009 | 928 | 3 | GC3 | ALII00000000; (14) |
| K50 | Urine / Kuwait / 2008 | 499 | 158 |  | OHJL00000000.1; (17) |
| LAC-4 | ND /Los Angeles/1997 | 447 | 10 |  | CP007712.1; (18) |
| M1 | ND/ Malaysia / 2009 | 195 | 2 | GC2 | LAIL00000000.1 |
| MDR-TJ | ND / Tianjin / ND | 369, 1837* | 2 | GC2 | CP003500.1; (19, 20) |
| MDR-ZJ06 | Blood/ Hangzhou / 2006 | 643* | 2 | GC2 | CP001937.2; (21) |
| Naval-81 | Blood / Betheseda, USA / 2006 | 928 | 3 | GC3 | AFDB00000000; (14) |
| OIFC098 | ND / Germany / 2003 | 391 | 10 |  | AMDF00000000; (14) |
| OIFC137 | Catheter tip / Washington / 2003 | 106 | 3 | GC3 | AFDK0000000; (14) |
| PR07 | Blood / Malaysia / ND | 734 | 239 |  | CP012035.1; (22) |
| R2090 | Rectal swab / Egypt / ND | 942 | 267 |  | LN868200.1; (23) |
| RBH3 | Endotracheal aspirate / Australia / 2002 | 781^Δ^ | 1 | GC1 | FBXD00000000; (3) |
| SDF | Human body louse / ND/ ND | NA | 17 |  | CU468230.2; (12) |
| TCDC | Blood / Taiwan / ND | 218* | 2 | GC2 | CP002522.2; (24) |
| TYTH-1 | Blood / Taiwan / 2006 | 455* | 2 | GC2 | CP003856.1; (25) |
| WC-A-694 | ND / Washington / 2008 | 928 | 3 | GC3 | AMTA00000000; (14) |
| ZW85-1 | Feces / China / ND | 378 | 639 |  | CP006768.1; (26) |
| 341 | Sputum / Malaysia / 2013 | 938 | 2 | GC2 | JQSD00000000 |
| 461 | Wound swab / Malaysia / 2013 | 195 | 2 | GC2 | LCTE00000000 |
| 863 | Sputum / Malaysia / 2014 | 938 | 2 | GC2 | LZTF01000000 |
| 1656-2 | Sputum / South Korea / 2004 | 423 | 2 | GC2 | CP001921.1; (27) |
| 9102 | Bronchial fluid/ Mexico/ ND | 231 | 1 | GC1 | CP023029.1; (28) |
| 5845 | Wound/ Mexico/ 2009 | 417 | 2 | GC2 | NZ_CP023034.1; (28) |
| 10042 | Secretion/ Mexico/ 2011 | 473 | 2 | GC2 | NZ_CP023026.1; (28) |

*^a^* ND – unknown

* Two *gdh-B* alleles found (*gdh-B-3* and *gdh-B-189*) which could correspond to more than one STs

^#^ Two *gdh-B* alleles found (*gdh-B-4* and *gdh-B-162*) which could correspond to more than one STs

^§^ Two *gdh-B* alleles found (*gdh-B-74* and *gdh-B-182*)

^Δ^Two *gdh-B* alleles found (*gdh-B-4* and *gdh-B-182*)

**Suppl. Table S1(B).**

| *A. nosocomialis* isolate | Source of isolate / Location / Year*^a^* | MLST | | Accession nos. / Reference(s) |
| --- | --- | --- | --- | --- |
|  |  | Oxford | Pasteur |  |
| 2010S01-197 | Respiratory/Taiwan/2010 | 1999* | 1272* | CP033561.1; (29) |
| 2010N17-248 | Blood/Taiwan/2010 | 1996* | 410 | CP033572.1; (29) |
| 2012C01-137 | Blood/Taiwan/2012 | 1343* | 217* | CP033557.1; (29) |
| 2014N23-120 | Blood/Taiwan/2014 | 1996* | 410* | CP033545.1; (29) |
| 2014S01-097 | Respiratory/Taiwan/2014 | 1996* | 410* | CP033550.1; (29) |
| 28F | ND/ Colombia /ND | 948 | 71 | CBSD000000000.2; (30) |
| 6411 | ND/Colombia/2012 | 1162 | 322 | CP010368 |
| Ab22222 | ND | 1066 | 71 | AKAR01000001 |
| AB6 | Blood/Guanzhou/2013 | 708 | 782 | PXNE01000001 |
| AB7 | Blood/Guanzhou/2014 | 708 | 782 | PXND01000001 |
| AB11 | Blood/Guanzhou/2014 | 2078 | 1264 | PXMZ01000001.1 |
| FDAARGOS-129 | Abscess/USA/2014 | 958 | 433 | CP014019.1; (31) |
| HJ14 | Kidney fluid/Zhejiang/2014 | 1343 | 217 | MADF01000001.1 |
| J1A | Sea water/Phillipine Sea/2016 | 715 | 768 | CP042994.1 |
| KAN01 | Sputum/Daejon, Korea/2015 | 1740 | 768 | CP038816.1 |
| KAN02 | Blood/Jeonju, Korea/2015 | 2078 | 1264 | CP036171.1 |
| LMG10619 | Sputum/Japan/ND | 1035 | 76 | BBSR01000001.1 |
| M2 | Hip infection /Ohio, USA/1996 | ND | ND | CP040105.1; (32, 33) |
| NCTC8102 | ND/Rhode Island, USA/1950 | 958 | 74 | CP029351.1; (34) |
| NIPH386 | Sputum/Czech Republic/1996 | 948 | 410 | KB849561.1 |
| NIPH2119 | Sputum/Rotterdam/1987 | 1035 | 76 | APOP01000001.1 |
| P020 | ND/Taiwan/2017 | 2078 | 1264 | APCE01000001.1 |
| SSA3 | Blood/Seoul/2013 | 958 | 433 | CP020588.1; (35) |
| T228 | ND/Bangkok/2010 | 1897 | 279 | JRUA01000001.1; (36) |
| UBA6823 | Metal/New York/ND (Metagenome sample) | 854^#^ | 1259* | DKEL01000001.1; (37) |

*^a^* ND – unknown

* alleles with less than 100% identity/imperfect hit

**REFERENCES FOR SUPPLEMENTARY TABLE S1:**

1. Hamidian M, Kenyon JJ, Holt KE, Pickard D, Hall RM. 2014. A conjugative plasmid carrying the carbapenem resistance gene blaOXA-23 in AbaR4 in an extensively resistant GC1 Acinetobacter baumannii isolate. J Antimicrob Chemother 69:2625–2628.

2. Hamidian M, Wick RR, Hartstein RM, Judd LM, Holt KE, Hall RM. 2019. Insights from the revised complete genome sequences of Acinetobacter baumannii strains AB307-0294 and ACICU belonging to global clones 1 and 2. Microb Genomics 5:e000298.

3. Hamidian M, Nigro SJ. 2019. Emergence, molecular mechanisms and global spread of carbapenem-resistant Acinetobacter baumannii. Microb Genomics 5:mgen.0.000306.

4. Hamidian M, Venepally P, Hall RM, Adams MD. 2017. Corrected Genome Sequence of Acinetobacter baumannii Strain AB0057, an Antibiotic-Resistant Isolate from Lineage 1 of Global Clone 1. Genome Announc 5:e00836-17.

5. Lean SS, Yeo CC, Suhaili Z, Thong KL. 2015. Whole-genome analysis of an extensively drug-resistant clinical isolate of Acinetobacter baumannii AC12: Insights into the mechanisms of resistance of an ST195 clone from Malaysia. Int J Antimicrob Agents 45:178–182.

6. Lean S-S, Yeo CC, Suhaili Z, Thong K-L. 2016. Comparative genomics of two ST 195 carbapenem-resistant Acinetobacter baumannii with different susceptibility to polymyxin revealed underlying resistance mechanism. Front Microbiol 6:1445.

7. Iacono M, Villa L, Fortini D, Bordoni R, Imperi F, Bonnal RJP, Sicheritz-Ponten T, De Bellis G, Visca P, Cassone A, Carattoli A. 2008. Whole-genome pyrosequencing of an epidemic multidrug-resistant Acinetobacter baumannii strain belonging to the European clone II group. Antimicrob Agents Chemother 52:2616–2625.

8. Weber BS, Ly PM, Irwin JN, Pukatzki S, Feldman MF. 2015. A multidrug resistance plasmid contains the molecular switch for type VI secretion in Acinetobacter baumannii. Proc Natl Acad Sci U S A 112:9442–9447.

9. Smith MG, Gianoulis TA, Pukatzki S, Mekalanos JJ, Ornston LN, Gerstein M, Snyder M. 2007. New insights into Acinetobacter baumannii pathogenesis revealed by high-density pyrosequencing and transposon mutagenesis. Genes Dev 21:601–614.

10. Zhu Y, Lu J, Zhao J, Zhang X, Yu HH, Velkov T, Li J. 2020. Complete genome sequence and genome-scale metabolic modelling of Acinetobacter baumannii type strain ATCC 19606. Int J Med Microbiol 310:151412.

11. Fournier P-E, Vallenet D, Barbe V, Audic S, Ogata H, Poirel L, Richet H, Robert C, Mangenot S, Abergel C, Nordmann P, Weissenbach J, Raoult D, Claverie J-M. 2006. Comparative genomics of multidrug resistance in Acinetobacter baumannii. PLoS Genet 2:e7.

12. Vallenet D, Nordmann P, Barbe V, Poirel L, Mangenot S, Bataille E, Dossat C, Gas S, Kreimeyer A, Lenoble P, Oztas S, Poulain J, Segurens B, Robert C, Abergel C, Claverie J-M, Raoult D, Médigue C, Weissenbach J, Cruveiller S. 2008. Comparative analysis of Acinetobacters: three genomes for three lifestyles. PLoS One 3:e1805.

13. Zhu L, Yan Z, Zhang Z, Zhou Q, Zhou J, Wakeland EK, Fang X, Xuan Z, Shen D, Li Q-Z. 2013. Complete genome analysis of three Acinetobacter baumannii clinical isolates in China for insight into the diversification of drug resistance elements. PLoS One 8:e66584.

14. Chan AP, Sutton G, DePew J, Krishnakumar R, Choi Y, Huang X-Z, Beck E, Harkins DM, Kim M, Lesho EP, Nikolich MP, Fouts DE. 2015. A novel method of consensus pan-chromosome assembly and large-scale comparative analysis reveal the highly flexible pan-genome of Acinetobacter baumannii. Genome Biol 16:143.

15. Krahn T, Wibberg D, Maus I, Winkler A, Pühler A, Poirel L, Schlüter A. 2015. Complete Genome Sequence of *Acinetobacter baumannii* CIP 70.10, a Susceptible Reference Strain for Comparative Genome Analyses. Genome Announc 3:e00850-15.

16. Farrugia DN, Elbourne LDH, Hassan K a, Eijkelkamp B a, Tetu SG, Brown MH, Shah BS, Peleg AY, Mabbutt BC, Paulsen IT. 2013. The complete genome and phenome of a community-acquired Acinetobacter baumannii. PLoS One 8:e58628.

17. Wibberg D, Salto IP, Eikmeyer FG, Maus I, Winkler A, Nordmann P, Poirel L, Schlüter A, Pühler A, Poirel L, Schlüter A. 2018. Complete genome sequencing of Acinetobacter baumannii strain K50 discloses the large conjugative plasmid pK50a encoding carbapenemase OXA-23 and extended-spectrum β-Lactamase GES-11. Antimicrob Agents Chemother 62:e00212-18.

18. Ou H-Y, Kuang SN, He X, Molgora BM, Ewing PJ, Deng Z, Osby M, Chen W, Xu HH. 2015. Complete genome sequence of hypervirulent and outbreak-associated Acinetobacter baumannii strain LAC-4: epidemiology, resistance genetic determinants and potential virulence factors. Sci Rep 5:8643.

19. Gao F, Wang Y, Liu YJ, Wu XM, Lv X, Gan YR, Song SD, Huang H. 2011. Genome sequence of Acinetobacter baumannii MDR-TJ. J Bacteriol 193:2365–2366.

20. Huang H, Yang Z-L, Wu X-M, Wang Y, Liu Y-J, Luo H, Lv X, Gan Y-R, Song S-D, Gao F. 2012. Complete genome sequence of Acinetobacter baumannii MDR-TJ and insights into its mechanism of antibiotic resistance. J Antimicrob Chemother 67:2825–2832.

21. Zhou H, Zhang T, Yu D, Pi B, Yang Q, Zhou J, Hu S, Yu Y. 2011. Genomic analysis of the multidrug-resistant Acinetobacter baumannii strain MDR-ZJ06 widely spread in China. Antimicrob Agents Chemother 55:4506–4512.

22. Izwan I, Teh LK, Salleh MZ. 2015. The genome sequence of Acinetobacter baumannii isolated from a septicemic patient in a local hospital in Malaysia. Genomics Data 6:128–129.

23. Krahn T, Wibberg D, Maus I, Winkler A, Nordmann P, Pühler A, Poirel L, Schlüter A. 2015. Complete Genome Sequence of the Clinical Strain Acinetobacter baumannii R2090 Carrying the Chromosomally Encoded Metallo-β-Lactamase Gene blaNDM-1. Genome Announc 3:e01008-15.

24. Chen CC, Lin YC, Sheng WH, Chen YC, Chang SC, Hsia KC, Liao MH, Li SY. 2011. Genome sequence of a dominant, multidrug-resistant Acinetobacter baumannii strain, TCDC-AB0715. J Bacteriol 193:2361–2362.

25. Liu C-C, Tang CY, Kuo H-Y, Lu C-W, Chang K-C, Liou M-L. 2013. The origin of Acinetobacter baumannii TYTH-1: a comparative genomics study. Int J Antimicrob Agents 41:318–324.

26. Wang X, Zhang Z, Hao Q, Wu J, Xiao J, Jing H. 2014. Complete Genome Sequence of Acinetobacter baumannii ZW85-1. Genome Announc 2:e01083-13.

27. Park JY, Kim S, Kim SM, Cha SH, Lim SK, Kim J. 2011. Complete Genome Sequence of Multidrug-Resistant Acinetobacter baumannii Strain 1656-2, Which Forms Sturdy Biofilm. J Bacteriol 193:6393–6394.

28. Salgado-Camargo AD, Castro-Jaimes S, Gutierrez-Rios R-M, Lozano LF, Altamirano-Pacheco L, Silva-Sanchez J, Pérez-Oseguera Á, Volkow P, Castillo-Ramírez S, Cevallos MA. 2020. Structure and Evolution of Acinetobacter baumannii Plasmids. Front Microbiol 11:1283.

29. Chen F-J, Huang W-C, Liao Y-C, Wang H-Y, Lai J-F, Kuo S-C, Lauderdale T-L, Sytwu H-K. 2019. Molecular Epidemiology of Emerging Carbapenem Resistance in Acinetobacter nosocomialis and Acinetobacter pittii in Taiwan, 2010 - 2014. Antimicrob Agents Chemother 63:e02007-18.

30. Barreto-Hernández E, Falquet L, Reguero MT, Mantilla JR, Valenzuela EM, González E. 2013. Draft Genome Sequences of Multidrug-Resistant Acinetobacter sp. Strains from Colombian Hospitals. Genome Announc 1:e00868-13.

31. Sichtig H, Minogue T, Yan Y, Stefan C, Hall A, Tallon L, Sadzewicz L, Nadendla S, Klimke W, Hatcher E, Shumway M, Aldea DL, Allen J, Koehler J, Slezak T, Lovell S, Schoepp R, Scherf U. 2019. FDA-ARGOS is a database with public quality-controlled reference genomes for diagnostic use and regulatory science. Nat Commun 10:3313.

32. Pehde B, Lizer N, Carruthers M. 2019. Complete Genome Sequence of the Nosocomial Pathogen *Acinetobacter nosocomialis* Strain M2. Microbiol Resour Announc 8:e00538-19.

33. Carruthers MD, Harding CM, Baker BD, Bonomo RA, Hujer KM, Rather PN, Munson RS. 2013. Draft Genome Sequence of the Clinical Isolate Acinetobacter nosocomialis Strain M2. Genome Announc 1:e00906-13.

34. Subhadra B, Surendran S, Lim BR, Yim JS, Kim DH, Woo K, Han K, Oh MH, Choi CH. 2019. Complete genome sequence and phylogenetic analysis of nosocomial pathogen Acinetobacter nosocomialis strain NCTC 8102. Genes and Genomics 41:1063–1075.

35. Yoon E-J, Kim JO, Yang JW, Kim HS, Lee KJ, Jeong SH, Lee H, Lee K. 2017. The blaOXA-23-associated transposons in the genome of Acinetobacter spp. represent an epidemiological situation of the species encountering carbapenems. J Antimicrob Chemother 72:2708–2714.

36. Loraine J, Heinz E, Soontarach R, Blackwell GA, Stabler RA, Voravuthikunchai SP, Srimanote P, Kiratisin P, Thomson NR, Taylor PW. 2020. Genomic and Phenotypic Analyses of Acinetobacter baumannii Isolates From Three Tertiary Care Hospitals in Thailand. Front Microbiol 11:548.

37. Parks DH, Rinke C, Chuvochina M, Chaumeil PA, Woodcroft BJ, Evans PN, Hugenholtz P, Tyson GW. 2017. Recovery of nearly 8,000 metagenome-assembled genomes substantially expands the tree of life. Nat Microbiol 2:1533–1542.
