# Supplementary figures and images for: Complete Genome Sequencing of Acinetobacter baumannii AC1633 and Acinetobacter nosocomialis AC1530 Unveils a Large Multidrug-Resistant Plasmid Encoding the NDM-1 and OXA-58 Carbapenemases
Source: mSphere. 2021 Jan 27;6(1):e01076-20. doi: 10.1128/mSphere.01076-20 (PMC7885321; doi:10.1128/mSphere.01076-20)

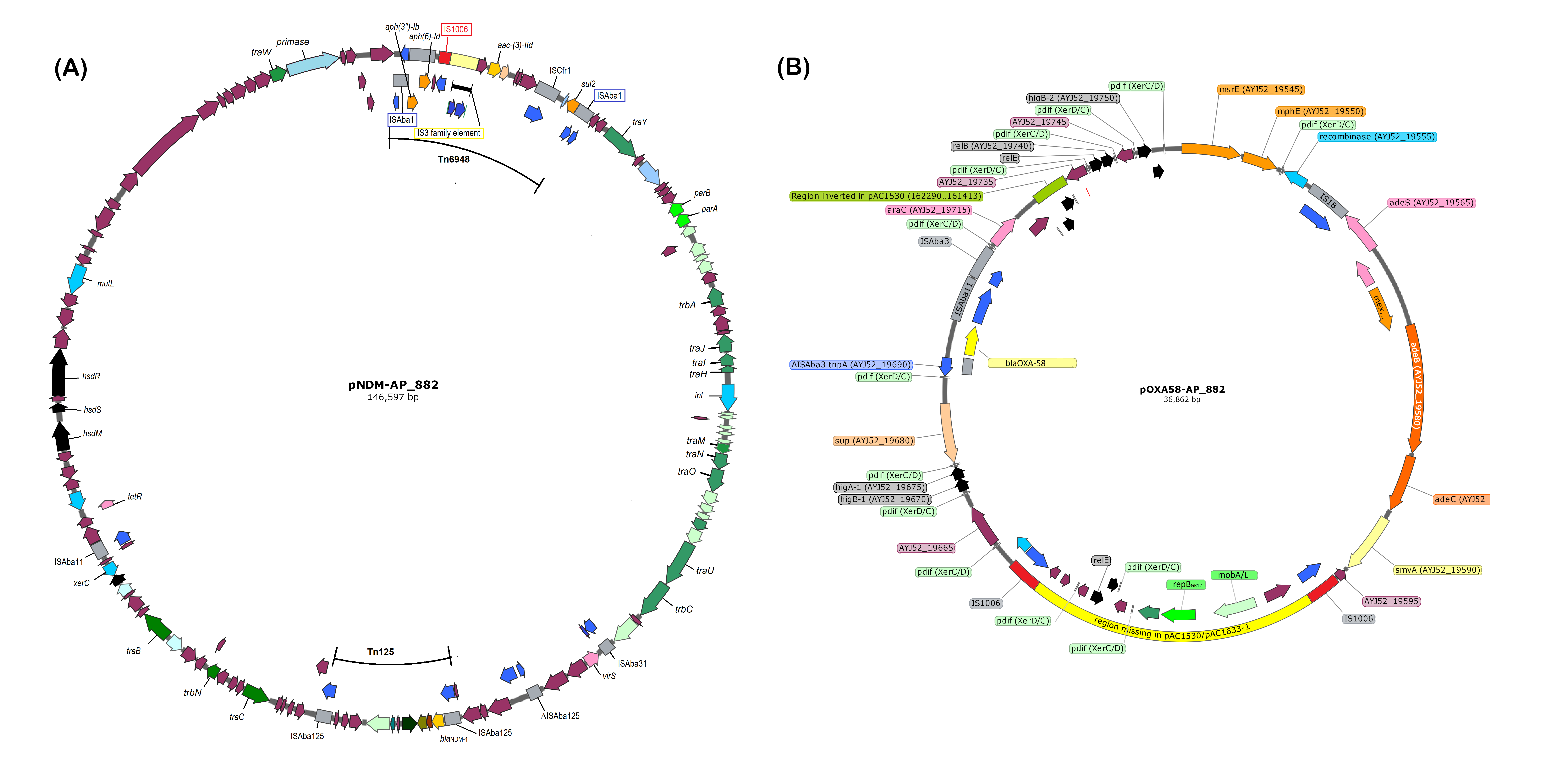

Supplement: FIG S1 [file mSphere.01076-20-sf001.tif]
